# The COF Space: Materials Features, Gas Adsorption, and Separation Performances Assessed by Machine Learning

**DOI:** 10.1021/acsmaterialslett.4c02594

**Published:** 2025-02-11

**Authors:** Gokhan Onder Aksu, Seda Keskin

**Affiliations:** Department of Chemical and Biological Engineering, Koc University, Rumelifeneri Yolu, Sariyer, 34450 Istanbul, Turkey

## Abstract

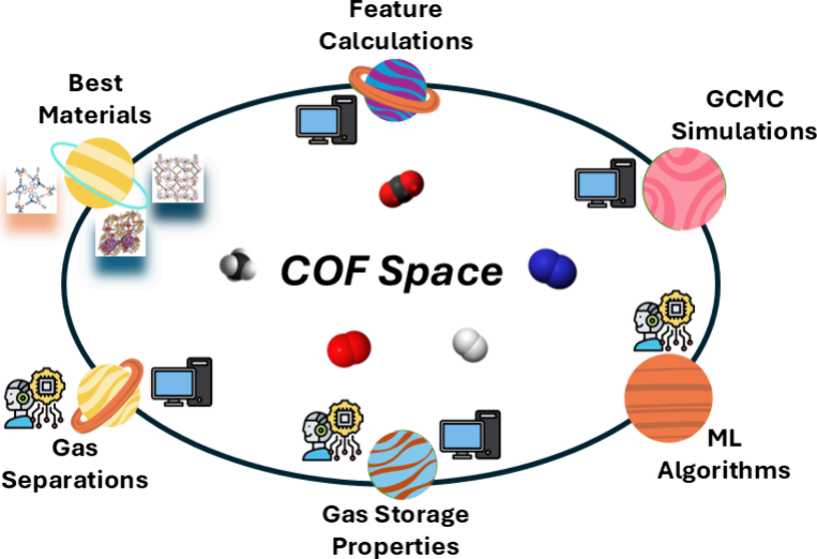

Covalent organic frameworks (COFs) are promising materials for gas adsorption; however, only a small number of COFs has been studied for a few types of gas separations to date. To unlock the full potential of the COF space, composed of 69 784 different types of materials, we studied the adsorption of five important gas molecules, CO_2_, CH_4_, H_2_, N_2_, and O_2_ in COFs at various pressures combining high-throughput molecular simulations and machine learning. Adsorbent performances of COFs were then explored for industrially critical separations, such as CO_2_/CH_4_, CO_2_/H_2_, CO_2_/N_2_, CH_4_/H_2_, CH_4_/N_2_, and O_2_/N_2_. The key structural and chemical properties of the most promising adsorbents were revealed. Our work offers the most extensive dataset produced for COFs in the literature composed of ∼4.3 million data points for all synthesized and hypothetical COFs’ structural, chemical, and energetic features; gas adsorption properties; and selectivities to facilitate the materials discovery.

Covalent organic frameworks (COFs) are mechanically, thermally, and chemically robust materials that have emerged as promising adsorbents for gas adsorption and separation thanks to their exceptional structural characteristics such as high porosities, large surface areas, and low densities.^1−5^ The number of experimentally synthesized and computationally designed COFs is increasing very rapidly. Experimentally discovered COFs were collected into two databases: the Computation-Ready Experimental (CoRE) COF database containing 1,242 structures,^6−8^ and the Clean, Uniform, Refined with Automatic Tracking from Experimental (CURATED) COF database, which originally includes 871 structurally optimized COFs.^9,10^ Smit’s group constructed the first hypothetical COF (hypoCOF) database having 69,840 computer-generated structures.^11,12^ Studying this large COF space using purely experimental methods to identify the most promising materials for the adsorption and separation of different gases is simply impossible.

High-throughput computational screening (HTCS) approach, which computes the gas adsorption properties of porous materials using molecular simulations, has been successful to accurately evaluate and identify the most useful candidates.^13−15^ For example, Tong et al.^16^ evaluated 46 COFs for CO_2_/CH_4_, CH_4_/H_2_, CO_2_/H_2_ separations using grand canonical Monte Carlo (GCMC) simulations, and then studied 187 CoRE COFs for noble gas separation.^6^ Lan et al.^17^ investigated 187 CoRE COFs by computing their iodine adsorption capacities, whereas 280 CoRE COFs were explored for CH_4_ storage by GCMC simulations.^7^ Smit’s group screened 296 CURATED COFs for CO_2_/N_2_ separation.^9^ HypoCOF databases were studied for CH_4_ deliverable capacities,^12,18^ and CO_2_/N_2_ separation.^11^ GCMC simulations were also used to compute H_2_ deliverable capacities of 449 CoRE COFs and 6,893 hypoCOFs.^19^ Our group performed HTCS of CoRE COFs, CURATED COFs, and hypoCOFs to examine CO_2_/H_2_,^20^ and H_2_S+CO_2_ separation from a natural gas mixture^21^ in addition to CH_4_ purification.^22^ All of these HTCS studies showed that COFs and hypoCOFs exhibit good separation performances, and many of them even outperform well-known zeolites and metal–organic frameworks (MOFs).

Studying the entire COF material spectrum consisting of thousands of different structures is computationally resource and time demanding. Therefore, HTCS has been recently combined with machine learning (ML) for accelerating the prediction of gas adsorption and separation capabilities of COFs.^23,24^ CH_4_ storage capacities of 69,839 hypoCOFs was explored by ML and using the chemical and structural features as inputs led to accurate predictions.^25^ A self-consistent ML approach was adapted to identify the top-performing hypoCOFs for CH_4_ storage.^26^ Active learning was used to screen hypoCOFs for CH_4_ storage and structures having high pore volumes achieved high CH_4_ delivery.^27^ HypoCOFs were studied by ML for CO_2_/N_2_ separation and results showed that imide, (keto)enamine hypoCOFs achieve efficient CO_2_ capture.^28^ ML models were developed to study CoRE COFs and hypoCOFs for CH_4_/H_2_ separation and narrow-pored hypoCOFs outperformed MOFs and zeolites in terms of selectivities.^29^ We recently utilized ML models trained on molecular fingerprints, digital representations of COFs based on their unique chemical properties, to study CO_2_/CH_4_ mixture separation.^30^

As this literature review shows, studies to date generally focused on either the synthesized or hypoCOF database and mostly examined two applications: CO_2_ separation or CH_4_ storage. The full potential of the COF space, composed of both synthetic and hypothetic materials, for the adsorption of different types of gas molecules is still unknown. Due to the lack of this important information, adsorbent performances of COFs for separating industrially important gases are also not available and are not comparable with other porous materials. To fill these critical gaps in the literature, in this work, we combined HTCS and ML approaches to assess the adsorption properties of both synthesized and hypothetical COFs, 69,784 different types of materials. We studied five important gas molecules CO_2_, CH_4_, H_2_, N_2_, and O_2_ at different pressures, 0.1, 1, 5, and 10 bar, to produce the largest gas adsorption dataset of COFs to date. Molecular simulations were performed for CoRE COFs and a representative subset of hypoCOFs to compute their gas uptakes and then used to train ML models that accurately estimate the gas uptake of any COF once the structural, chemical, and energetic features of the material are given. Uptake predictions were used to evaluate COFs’ adsorbent performances for six industrially important gas separations, CO_2_/CH_4_, CO_2_/H_2_, CO_2_/N_2_, CH_4_/H_2_, CH_4_/N_2_, and O_2_/N_2_ which represent natural gas purification, syngas separation, flue gas separation, hydrogen purification, natural gas upgrading and air separation, respectively.

Our work offers the most extensive dataset produced for COFs to date in the literature, composed of ∼4.3 million data points: ∼1.2 million data points for COFs’ chemical, structural, and energetic features; ∼1.4 million data points for gas uptakes at various pressures; and ∼1.7 million data for selectivities. Detailed analysis of this large dataset using ML identified the key structural and chemical properties of COFs for achieving high uptakes and selectivities. Through comparison with MOFs and zeolites, we unlocked the full potential of the COF space for various gas adsorption and separation applications. These results will significantly accelerate the materials’ discovery by directing future experimental efforts and time to the most promising materials.

Figure 1 provides a schematic representation of our computational methodology combining molecular simulations with ML to evaluate the gas adsorption properties of CoRE COFs and hypoCOFs. We worked on the seventh version of CoRE COF^6^ and the latest version of hypoCOF database,^12^ comprising 1,242 synthesized COFs and 69,840 hypoCOFs. Initial filtering of databases as explained in detail in the Supporting Information (SI) resulted in a final set of 1,060 CoRE COFs and 68,724 hypoCOFs. Using Zeo++ software (version 0.3),^31^ we computed structural properties of COFs; pore limiting diameter (PLD), the largest cavity diameter (LCD), accessible surface area (*S*_acc_), and porosity (ϕ). RASPA software^32^ was used to compute gas adsorption at pressures of 0.1, 1, 5, and 10 bar, at 298 K. All details of GCMC simulations are given in the SI.

**Figure 1:**
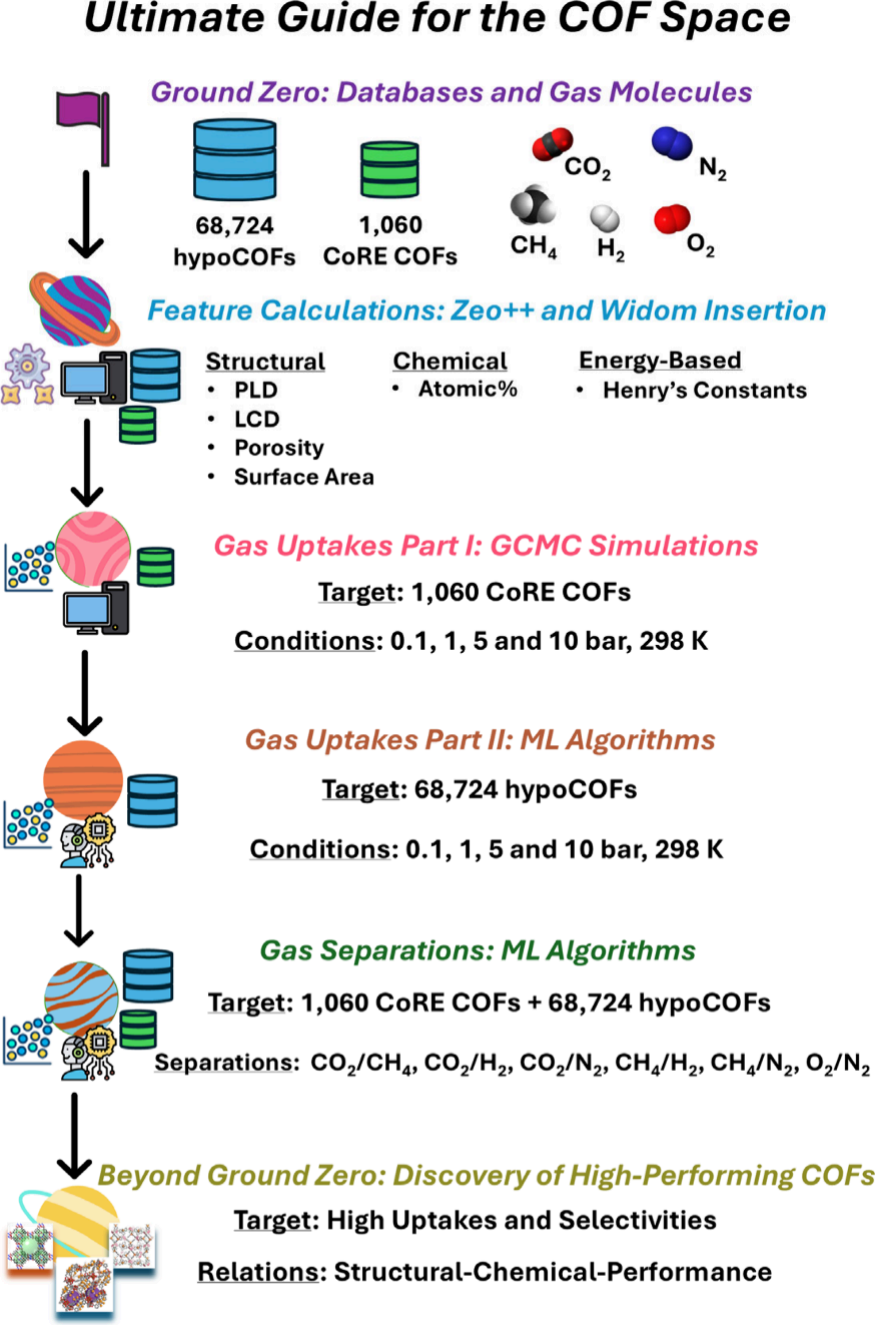
The computational workflow that we designed to study gas adsorption and separation performances of the COF Space.

Considering the extensive size of the COF space, performing molecular simulations for each material would require an enormous computational time and resources. To overcome this challenge, we developed ML models using the CO_2_, CH_4_, H_2_, N_2_, and O_2_ adsorption results obtained from molecular simulations as the target data and structural, chemical, energy-based descriptors of COFs (listed in Table S1) as the input data. The feature set included four structural descriptors (PLD, LCD, *S*_acc_, and ϕ) calculated by using Zeo++ software (version 0.3); seven chemical descriptors (percentages of carbon, hydrogen, nitrogen, oxygen, halogens, ametals, and metalloids in the COFs) extracted from the crystallographic information files obtained from the corresponding COF databases; and an energy-based descriptor (Henry’s coefficients for CO_2_, CH_4_, H_2_, N_2_, and O_2_). The tree-based pipeline optimization tool (TPOT)^33^ within the automated ML framework was utilized to identify the most appropriate algorithms and optimize their hyperparameters. In TPOT, regression algorithms from the scikit-learn library were employed for model selection. A stratified sampling approach was adopted to ensure a consistent distribution of features across the training and test datasets, with 80% of the data allocated for training and 20% for testing. To prevent overfitting, a 5-fold cross-validation was applied. Twenty ML models were developed to predict CO_2_, CH_4_, H_2_, N_2_, and O_2_ adsorption of the COF space at four different pressures and evaluated by calculating the statistical accuracy metrics including the coefficient of determination (*R*^2^), mean absolute error (MAE), root-mean-square error (RMSE), and Spearman’s rank correlation coefficient (SRCC), as listed in Table S2. All details of ML models are given in the SI, input and target data used in training these models are available at https://github.com/gokhanonderaksu/COFSpace.

We then focused on adsorption-based gas separation performances of COFs by computing their ideal selectivities, the ratios of the desired gas uptake to the undesired gas uptake, for CO_2_/CH_4_, CO_2_/H_2_, CO_2_/N_2_, CH_4_/H_2_, CH_4_/N_2_, and O_2_/N_2_ separations. Top-performing adsorbents offering exceptional selectivity for multiple gas separations were identified by ranking ML-predicted selectivities of COFs at 1 bar, and these materials were examined at the molecular level to reveal their key structural and chemical characteristics.

## Understanding CoRE COFs

**Figure 2:**
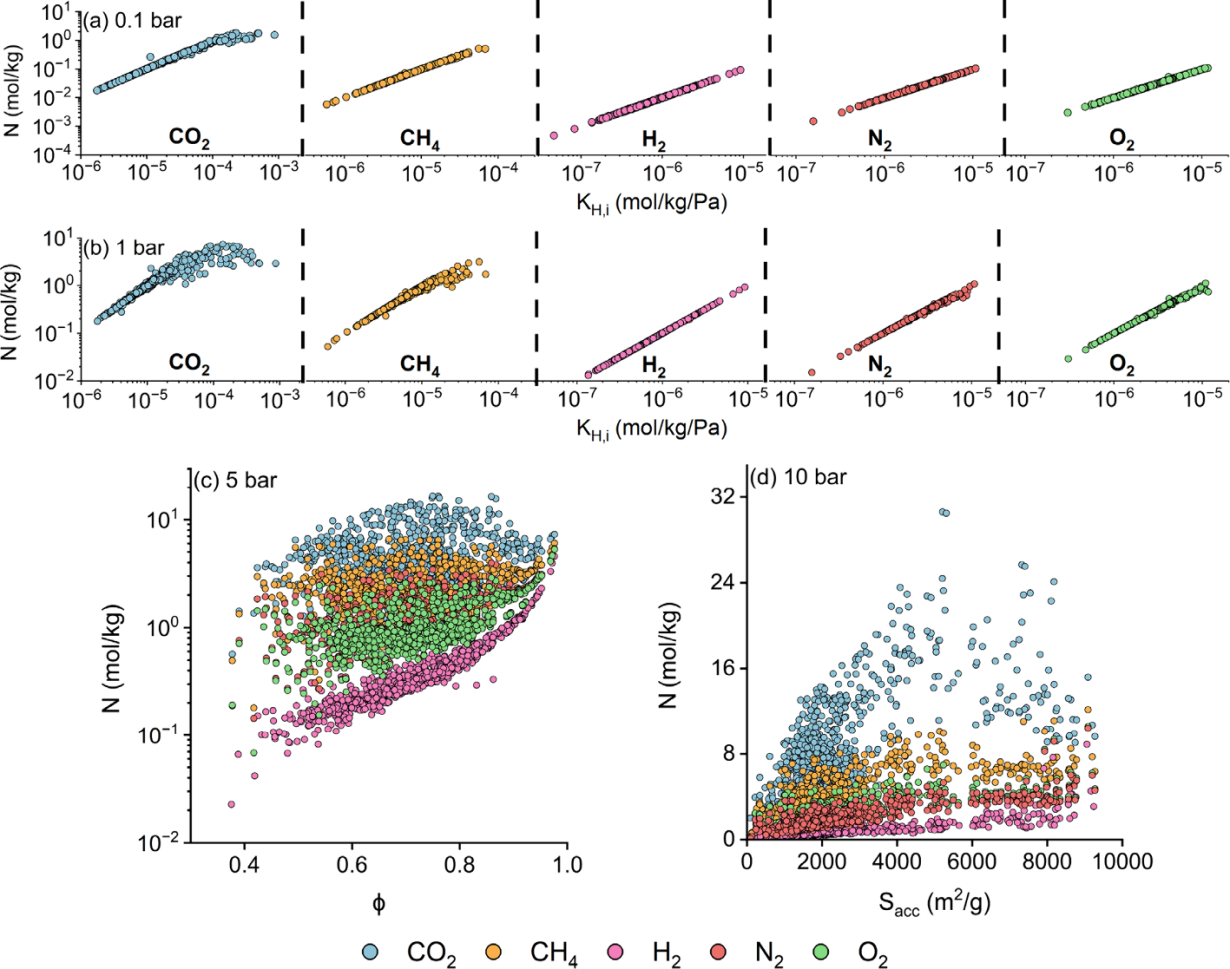
Simulated CO_2_, CH_4_, H_2_, N_2_, and O_2_ uptake data (N) for 1,060 CoRE COFs with respect to the most correlated materials’ features at (a) 0.1, (b) 1, (c) 5, and (d) 10 bar.

Figure 2 shows simulated CO_2_, CH_4_, H_2_, N_2_, and O_2_ adsorption data of 1,060 CoRE COFs with respect to their correlated features obtained from the Pearson correlation heat maps given in Figures S1–S5 at various pressures. The Henry’s constants of all gases (*K*_H,i_) consistently show the highest correlations with the adsorbed gas amounts at 0.1 and 1 bar, since *K*_H,i_ indicates the strength of interactions between gases and COFs at low pressures. Figure 2(a) shows that CO_2_ has the highest uptakes (0.02–1.79 mol/kg), followed by CH_4_ (0.01–0.52 mol/kg), O_2_ (3 × 10^–3^–0.11 mol/kg), N_2_ (1.6 × 10^–3^–0.11 mol/kg), and H_2_ (4.8 × 10^–4^–0.09 mol/kg) at 0.1 bar. Similarly, uptakes are correlated to *K*_H,i_ at 1 bar. Figure 2(b) illustrates that COFs have the highest uptakes for CO_2_ (0.18–7.42 mol/kg) thanks to the presence of strong Van der Waals and Coulombic interactions, followed by CH_4_ (0.05–3.11 mol/kg), O_2_ (0.03–1.11 mol/kg), N_2_ (0.02–1.07 mol/kg), and H_2_ (4.6 × 10^–3^–0.93 mol/kg). As the pressure increases, gas molecules start to fill the available void spaces in COFs, and thus, porosity and surface area become critical for determining the adsorption amounts.

Figure 2(c) shows that CO_2_ uptakes at 5 bar (0.57–16.51 mol/kg) exhibit a linear relation with porosity for COFs having porosities <0.7, while CH_4_ (0.18–6.60 mol/kg), O_2_ (0.14–5.36 mol/kg), N_2_ (0.07–5.31 mol/kg), and H_2_ (0.02–4.57 mol/kg) uptakes demonstrate strong linear correlations within the entire porosity ranges. At 10 bar, in Figure 2(d), uptakes of CO_2_ (0.65–30.62 mol/kg), CH_4_ (0.26–12.17 mol/kg), O_2_ (0.28–10.58 mol/kg), N_2_ (0.12–10.43 mol/kg), and H_2_ (0.05–8.88 mol/kg) and surface areas of COFs are very strongly correlated. These results agree with previous findings on H_2_ adsorption in MOFs, where surface area and porosity were reported as dominating factors defining the adsorbed amounts at intermediate and high pressures.^34^

The next step was developing ML models capable of accurately and rapidly predicting gas uptakes of the COF space without performing computationally demanding molecular simulations. For this purpose, we trained ML models, named as “CoRE ML models”, using the structural, chemical, energy-based descriptors of 1,060 CoRE COFs as the input data and simulated gas uptakes as the target data. Details of CoRE ML models are listed in Tables S3–S6. The accuracies of these models were assessed by comparing ML-predicted gas uptakes with the simulated ones in Figure S6–S9 and by calculating the accuracy metrics given in Table S7. For all gases, both *R*^2^ and SRCC values of the test sets generally exceed 0.9 at all conditions, indicating the accuracy of “CoRE ML models” for predicting CO_2_, CH_4_, H_2_, N_2_, and O_2_ uptakes of CoRE COFs.

## From CoRE to Universal: HypoCOFs

To confidently use our CoRE ML models for the COF space, transferring them to an unseen hypoCOF dataset is critical. To do this, we first selected a representative subset of 6,872 hypoCOFs, 10% of the entire hypoCOFs, that have similar feature distributions to hypoCOF space in terms of surface area, porosity, and *K*_H,i_ as shown in Figure S10, so that our models can maintain their prediction accuracy across all possible feature combinations. However, CoRE ML models were unable to accurately predict CO_2_, CH_4_, and O_2_ adsorption in hypoCOFs at some pressures as discussed in detail in SI and represented in Figures S11–S15 and Table S8. Therefore, we retrained CoRE ML models using an additional dataset of 563 hypoCOFs, named these models as “CoRE+Hypo” ML models, and showed their transferability to hypoCOFs, as we discussed in detail in the SI (Tables S9–S14, Figures S16–23).

**Figure 3:**
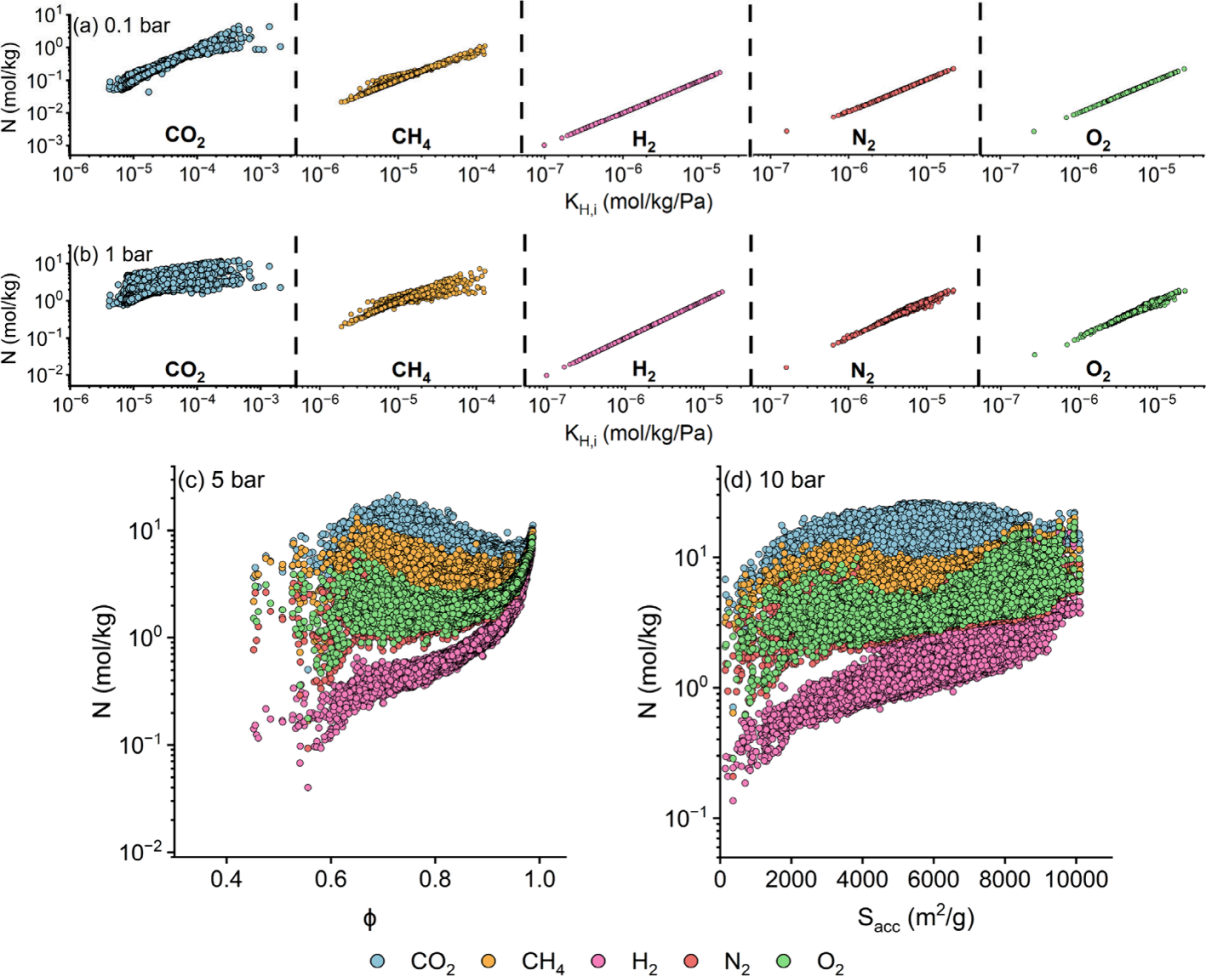
ML-predicted CO_2_, CH_4_, H_2_, N_2_, and O_2_ adsorption data (N) for 68,724 hypoCOFs with respect to the most correlated materials’ features at (a) 0.1, (b) 1, (c) 5, and (d) 10 bar.

These CoRE+Hypo ML models were then used to assess gas uptakes of 68,724 previously unexamined hypoCOFs. Figure 3 presents ML-predicted gas adsorption data as a function of K_H,i_, porosities, and surface areas at different pressures. Figure 3(a) demonstrates that CO_2_, CH_4_, O_2_, N_2_, and H_2_ uptakes in hypoCOFs range from 0.03 to 4.48, 0.01 to 1.10, 2.7 × 10^–3^ to 0.22, 2.6 × 10^–3^ to 0.21, and 1 × 10^–3^ to 0.17 mol/kg, respectively, at 0.1 bar. Same with CoRE COFs, *K*_H,i_ remains the primary factor in determining gas uptakes of hypoCOFs. There are 42, 36, 343, 319, and 322 hypoCOFs achieving higher CO_2_ (>1.79 mol/kg), CH_4_ (>0.52 mol/kg), O_2_ (>0.11 mol/kg), N_2_ (>0.11 mol/kg), and H_2_ (>0.09 mol/kg) uptakes compared to CoRE COFs having the highest uptakes. Figure 3(b) shows that ML-predicted CO_2_, CH_4_, O_2_, N_2_, and H_2_ uptakes range between 0.55 and 12.07, 0.05 and 7.14, 0.04 and 1.87, 0.02 and 1.91, and 0.01 and 1.73 mol/kg, respectively, at 1 bar. 89, 54, 325, 340, and 300 hypoCOFs were identified to surpass the highest uptakes observed in CoRE COFs for CO_2_ (>7.42 mol/kg), CH_4_ (>3.1 mol/kg), O_2_ (>1.11 mol/kg), N_2_ (>1.07 mol/kg), and H_2_ (>0.93 mol/kg), respectively.

Figure 3(c) highlights increased gas uptakes of COFs for CO_2_ (0.50–21.04 mol/kg), CH_4_ (0.17–13.04 mol/kg), O_2_ (0.18–8.71 mol/kg), N_2_ (0.09–8.60 mol/kg), and H_2_ (0.04–8.48 mol/kg) having high porosities (>0.7). There are 62, 409, 325, 309, and 299 hypoCOFs surpassing CoRE COF benchmarks for CO_2_ (>16.51 mol/kg), CH_4_ (>6.60 mol/kg), O_2_ (>5.36 mol/kg), N_2_ (>5.31 mol/kg), and H_2_ (>4.57 mol/kg) at 5 bar. Figure 3(d) shows that CO_2_ (0.71–26.1 mol/kg), CH_4_ (0.64–19.94 mol/kg), O_2_ (0.25–18.44 mol/kg), N_2_ (0.21–18.24 mol/kg), and H_2_ (0.14–17.02 mol/kg) uptakes are high in COFs having surface areas >5,000 m^2^/g. No hypoCOF achieved higher uptakes than the highest uptake of CoRE COFs for CO_2_ (>30.62 mol/kg). There are 351, 326, 340, and 310 hypoCOFs surpassing the highest uptakes of CoRE COFs for CH_4_ (>12.2 mol/kg), O_2_ (>10.6 mol/kg), N_2_ (>10.4 mol/kg), and H_2_ (>8.9 mol/kg) at 10 bar. Higher gas uptakes of hypoCOFs compared to CoRE COFs can be attributed to the diversity in their structural characteristics, particularly large surface areas and porosities since 89% of hypoCOFs have surface areas >5,000 m^2^/g and pore sizes >50 Å, whereas only 5% of CoRE COFs exhibit these characteristics.

Overall, hypoCOFs achieve higher gas uptakes than CoRE COFs at different pressures except for CO_2_ uptakes at 10 bar. We also observed that structural features of the hypoCOFs leading to high gas uptakes can differ based on the gas type. For example, 20 hypoCOFs with the highest CO_2_ uptakes (25.4–26.1 mol/kg) at 10 bar have mediocre pores (LCD: 8.4–11.7 Å), porosities (0.75–0.81) and large surface areas (5,148–6,134.6 m^2^/g). The 20 hypoCOFs with the highest CH_4_ (16.6–19.9 mol/kg), H_2_ (13.2–17 mol/kg), N_2_ (14.8–18.2 mol/kg), and O_2_ (15–18.5 mol/kg) uptakes tend to have large pores (LCD > 50 Å), and very high porosities (>0.97) and surface areas (>8,000 m^2^/g). We also note that although the hypoCOF database was constructed using the linker and topology information of the experimental COF structures, future efforts to synthesize and test gas adsorption properties of these hypothetical materials would be very important.

## Assessing the COF Space for Gas Separations

**Figure 4:**
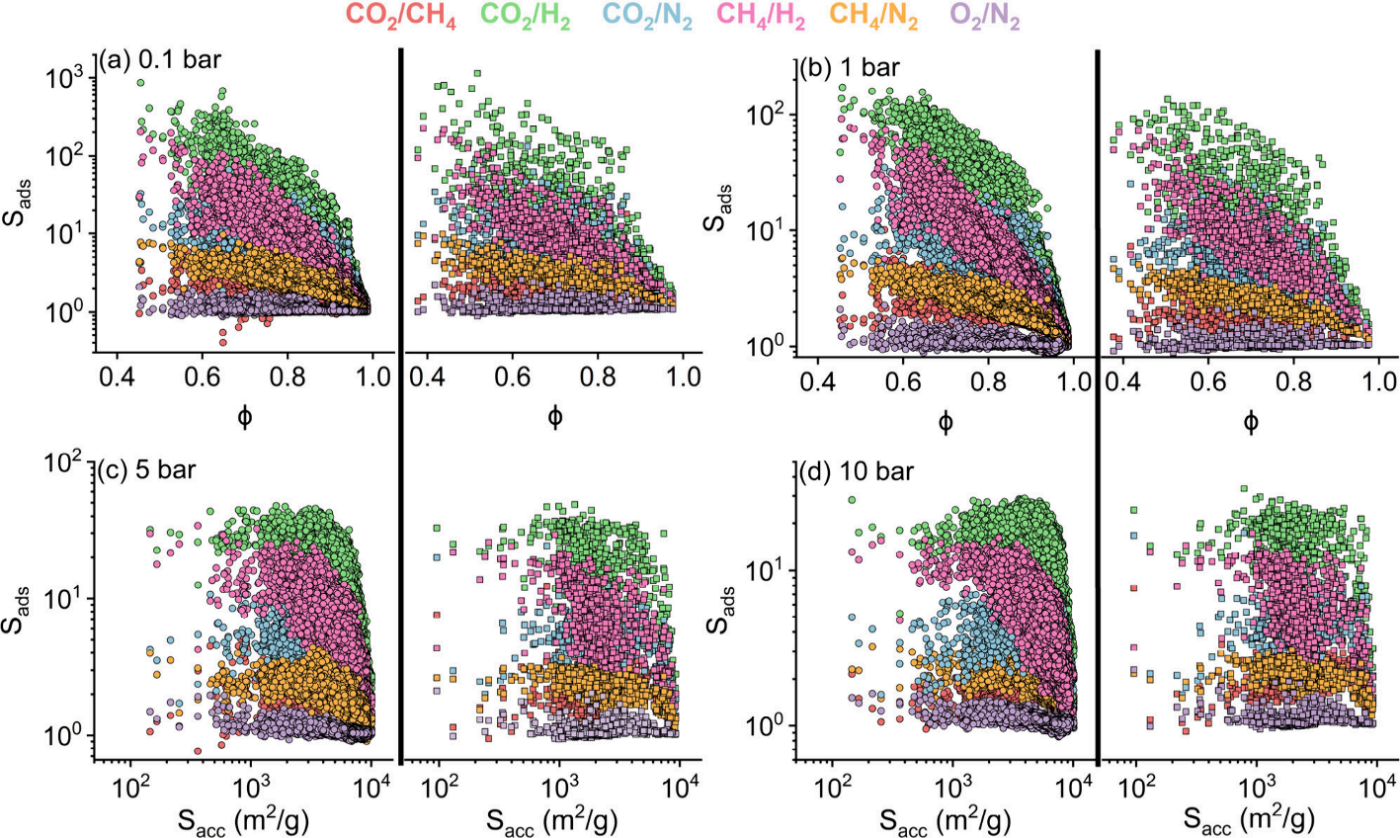
Selectivities of the COF space for CO_2_/CH_4_, CO_2_/H_2_, CO_2_/N_2_, CH_4_/H_2_, CH_4_/N_2_, and O_2_/N_2_ separations. Selectivities of 68,724 hypoCOFs (left panel) and 1,060 CoRE COFs (right panel) were computed at (a) 0.1, (b) 1, (c) 5, and (d) 10 bar.

The enormous adsorption data of hypoCOFs presented in Figure 4 was produced by ML models only in a few minutes as an extremely efficient alternative to performing molecular simulations that require many months for each COF and gas molecule. Since this large adsorption data was produced for the first time for the COF space, we compared them with other porous materials in the SI and concluded that COFs achieve comparable or even superior gas uptakes than MOFs. We then assessed the potential of the COF space by computing ideal selectivities for different gas separations. Figure 4 illustrates COFs’ selectivities of COFs as a function of the materials’ surface areas and porosities across different pressures. The selectivity trends follow CO_2_/H_2_ > CH_4_/H_2_ > CO_2_/N_2_ > CO_2_/CH_4_ ≈ CH_4_/N_2_ ≫ O_2_/N_2_. All COFs were CO_2_ selective, and the highest selectivities were observed for CO_2_/H_2_ separation (up to 863 at 0.1 bar and 29 at 10 bar) since CO_2_ (H_2_) was the most strongly (weakly) adsorbed component. The second application that COFs are promising is CH_4_/H_2_ separation, with selectivities up to 202 at 0.1 bar and 16 at 10 bar. COFs can be promising for CO_2_/N_2_ and CO_2_/CH_4_ separations but not for the O_2_/N_2_ separation due to low selectivities of 0.8–2.3 arising from similar molecular properties of these gases. As pressure increases, lower selectivities were observed since entropic effects favor the smaller gas molecules to fill the void spaces.

Figure 4(b) shows that the selectivities of CO_2_/H_2_, CH_4_/H_2_, CO_2_/N_2_, CO_2_/CH_4_, CH_4_/N_2_, and O_2_/N_2_ vary between 1.2–171, 1.2–91.9, 1.1–35, 1–17.5, 1.1–5.8, and 0.9–2.3 at 1 bar. The decreasing selectivity trend continues at 5 and 10 bar in Figures 4(c-d). Selectivities of CoRE and hypoCOFs were generally similar for all gas separations. For example, only 6 hypoCOFs achieved higher selectivities than the maximum selectivity observed for CoRE COFs for CO_2_/H_2_ separation (>135). Overall, at all conditions, we observed that both CoRE COFs and hypoCOFs achieving high CO_2_/H_2_, CH_4_/H_2_, CO_2_/N_2_, CO_2_/CH_4_, and CH_4_/N_2_ selectivities have mostly narrow pores (LCD < 10 Å), low porosities (<0.7), and mediocre surface areas (<2,500 m^2^/g). Detailed comparison of COF adsorbents with MOFs and zeolites is given in the SI and we inferred that many COFs outperform zeolites and MOFs in terms of selectivity, specifically for CO_2_/H_2_ separation.

**Figure 5:**
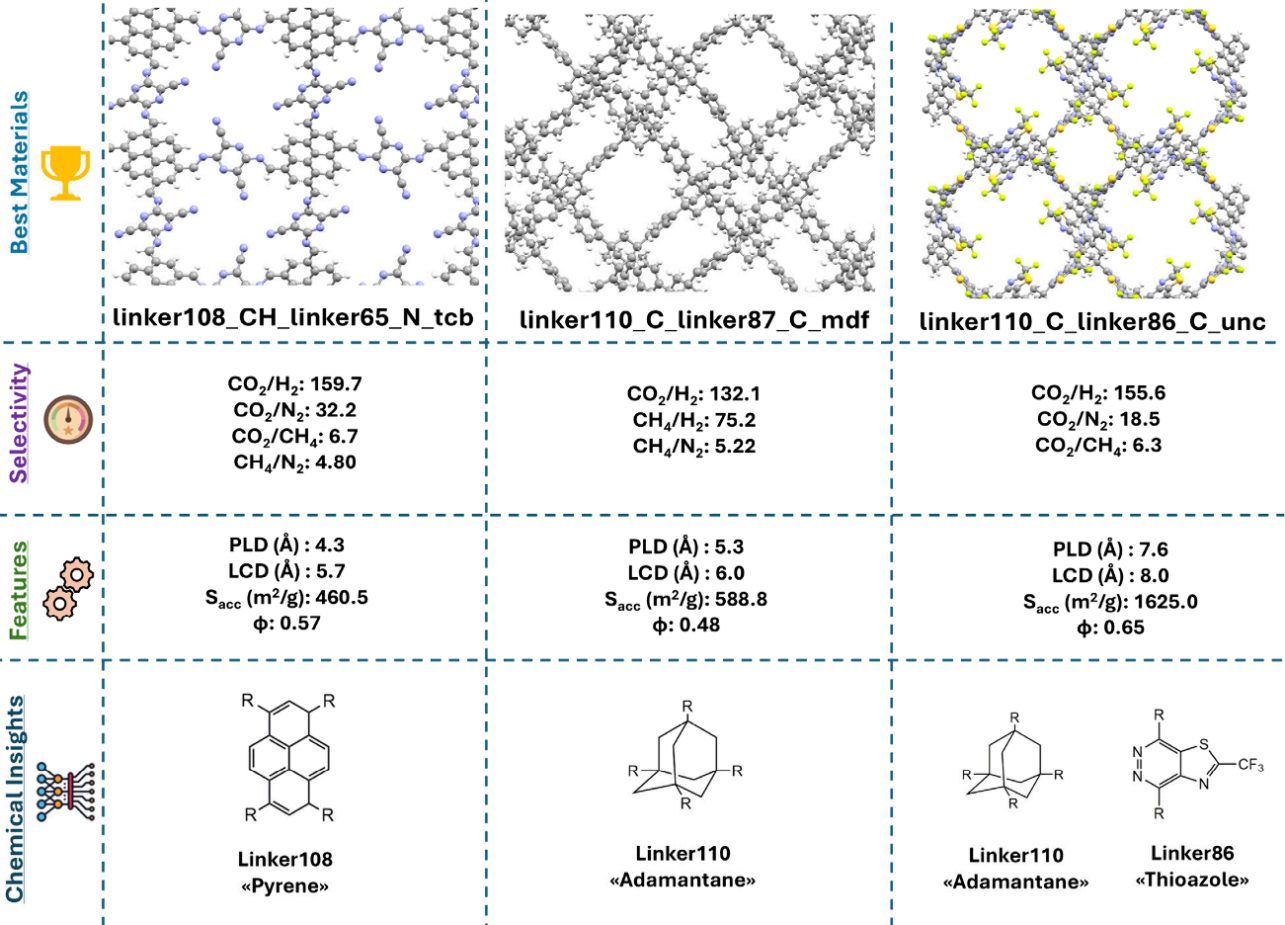
Structural and chemical analyses of three hypoCOFs identified as top materials for at least three different gas separations.

Figure 5 highlights the structural and chemical properties of the three most promising COFs. These materials have narrow pores (PLD: 4.3–7.6, LCD: 5.7–8 Å), and low surface areas (<2,000 m^2^/g) compared to most hypoCOFs. linker108_CH_linker65_N_tcb_interp_2 is the only common top-performing material identified for four different separations. This COF offers high CO_2_/H_2_ and CO_2_/N_2_ selectivities since it has pyrene-based linkers, symmetrical polycyclic aromatic hydrocarbons composed of four benzene rings, which provide favorable adsorption sites for CO_2_.^35^ linker110_C_linker87_C_mdf exhibits high selectivities for CO_2_/H_2_, CH_4_/H_2_, and CH_4_/N_2_ separations, while linker110_C_linker86_C_unc was identified as promising for all three CO_2_ separations. For both materials, the incorporation of adamantane-based linker groups promotes CO_2_ adsorption,^11,29^ whereas the presence of thiazole groups in linker86 further enhance CO_2_ affinity.^8,20^ This analysis shows that the best COF adsorbents have narrow pore structures (<8 Å), low porosities (0.48–0.65), and linkers having pyrene, adamantane, and thiazole groups.

In this study, we present the COF Space, the most extensive adsorption dataset dedicated to both synthesized and hypothetical COFs. Our results revealed that hypoCOFs demonstrate exceptional adsorption capacities, particularly for CO_2_ (up to 12.1 mol/kg), CH_4_ (up to 7.1 mol/kg), and H_2_ (up to 1.73 mol/kg) at 1 bar, surpassing the performance of synthesized COFs, MOFs, and zeolites. The hypoCOF space offers remarkable selectivities for CO_2_/H_2_, CH_4_/H_2_, CO_2_/N_2_, and CO_2_/CH_4_ separations, often outperforming experimentally studied COFs and zeolites, though not compatible to several MOFs. By unlocking the potential of COFs for selective adsorption of several gases and providing molecular insights into the key characteristics of the most selective adsorbents, our results will accelerate the design of novel COFs and their implementation in practical applications.

## Data Availability

All simulated and ML-predicted data are available together with ML scripts in https://github.com/gokhanonderaksu/COFSpace.

## Supporting Information Available

Computational details of molecular simulations and development of ML models; correlation matrix between structural, chemical, energy-based properties and gas uptakes of trained COF set; comparisons of the simulated and ML-predicted CO_2_, CH_4_, H_2_, N_2_, and O_2_ uptakes of train and test sets used in CoRE and CoRE+Hypo ML models; the box plot data distributions of unseen subset and whole database of hypoCOFs; comparisons of the simulated and ML-predicted CO_2_, CH_4_, H_2_, N_2_, and O_2_ uptakes of unseen hypoCOFs used in CoRE and CoRE+Hypo ML models; comparisons of COF space with the literature for gas adsorption and separations (PDF)
