# Supplementary material for: The COF Space: Materials Features, Gas Adsorption, and Separation Performances Assessed by Machine Learning
Source: ACS Mater Lett. 2025 Feb 11;7(3):954–60. doi: 10.1021/acsmaterialslett.4c02594 (PMC11881133; doi:10.1021/acsmaterialslett.4c02594)
Supplement: Supplementary file 1 — tz4c02594_si_001.pdf [file tz4c02594_si_001.pdf]

Supporting Information

for

The COF Space: Materials Features, Gas Adsorption, and Separation Performances Assessed by Machine Learning

Gokhan Onder Aksu, Seda Keskin*

Department of Chemical and Biological Engineering, Koç University, Rumelifeneri Yolu, Sariyer, 34450, Istanbul, Turkey

Submitted to *ACS Materials Letters*

1. Details of molecular simulations……………………………………………………….………2
2. Details of machine learning (ML)…………………………………………………….... 3
3. Development and transferability test of CoRE ML Models …………………………......11
4. Development and transferability test of CoRE+Hypo ML Models …………………..20
5. Comparison of the COF Space with Other Porous Materials……………………...….30

*Corresponding author. E-mail: skeskin@ku.edu.tr Phone: +90 (212) 338-1362.

1. Details of molecular simulations:

We used Zeo++ software (version 0.3)[1] to compute the structural properties of each COF, including pore limiting diameter (PLD), the largest cavity diameter (LCD), accessible surface area (S_{acc}), and porosity (ϕ). The S_{acc} values were calculated with a probe radius of 1.82 Å, representative of N_2 molecule. To ensure the adsorption of all gas molecules that we studied, materials with PLDs below 3.8 Å (kinetic diameter of the largest molecule, CH_4) and S_{acc} values of zero were excluded. Additionally, CoRE COFs containing metals were omitted to maintain the consistency with the hypoCOF database, which consists solely of metal-free structures. This filtering resulted in the selection of 1,081 CoRE COFs and 69,828 hypoCOFs for further analysis. The charge equilibration method, Q_{eq},[2] available in the RASPA simulation software,[3] was used to assign the partial point charges of COFs to compute the electrostatic interactions between polar gas molecules and frameworks. 21 CoRE COFs and 1,104 hypoCOFs for which charge assignment could not be performed due to their very large unit cells were excluded. This initial filtering resulted in a final set of 1,060 CoRE COFs and 68,724 hypoCOFs.

We employed the RASPA software[3] to compute the gas adsorption amounts in COFs at pressures of 0.1, 1, 5, and 10 bar, and a fixed temperature of 298 K. GCMC simulations were performed for all 1,060 CORE COFs and a representative set of 6,872 hypoCOFs selected to adequately represent the entire hypoCOF material space as discussed in detail in the following section. To model the interactions between COFs and gas molecules, as well as gas-gas interactions, we utilized Lennard-Jones (LJ) 12-6 and Coulombic potentials, and used the DREIDING force field[4] for the framework atoms.

CO_2 was modeled as a three-site linear, rigid molecule with a C–O bond length of 1.16 Å and partial point charges located at the center of each site.[5] CH_4 was modeled as a single-site molecule.[6] A single-site spherical model with the LJ 12-6 potential was used to model H_2.[7] N_2 was represented as a three-site rigid molecule with N atoms at the two sites and the center of mass with partial charges at the third site.[8] Similar to N_2, O_2 was modeled as a three-site molecule including two sites with O atoms and the third one with the center of mass.[9] The success of the molecular simulations using these potentials for gas molecules and COFs was previously shown by the good agreement between the simulation results and experimentally measured gas adsorption properties of COFs in different studies.[10-14] The dimensions of the simulation box were extended to at least 28 Å to accommodate a cut-off radius of 14 Å. Electrostatic interactions between CO_2, N_2, O_2 molecules and COFs were computed using the Ewald summation.[15] In GCMC simulations, we used 10,000 cycles for initialization and 20,000

cycles for taking the ensemble averages. To quantify the strength of interactions between gas molecules and COFs, we calculated the Henry's constants of gas molecules by using the Widom insertion method with 50,000 cycles.[16] We note that the accuracy of our molecular simulations was previously validated by comparing the simulation results with the experimentally measured single-component gas adsorption in various COFs.[10-12]

2. Details of machine learning (ML):

Given the vast diversity of COF structures, conducting molecular simulations for each individual material would necessitate substantial computational resources and extensive processing time. To tackle this challenge, we developed ML models using the molecular simulation data for CO_2, CH_4, H_2, N_2, and O_2 adsorption together with the computed structural, chemical, and energetic features of COFs. As shown in **Table S1**, the feature set included four structural descriptors (PLD, LCD, S_{acc}, and ϕ) calculated by using Zeo++ software (version 0.3); seven chemical descriptors (percentages of carbon, hydrogen, nitrogen, oxygen, halogens, ametals, and metalloids in the COFs) extracted from the crystallographic information files obtained from the corresponding COF databases;[17, 18] and an energy-based descriptor (Henry's coefficients for CO_2, CH_4, H_2, N_2, and O_2).

Table S1. The list of descriptors used to construct ML models.

Descriptor Type	Descriptors
Structural (4 descriptors)	• Largest Cavity Diameter (Å), LCD<br>• Pore Limiting Diameter (Å), PLD<br>• Accessible Surface Area (m^2/g), S_{acc}<br>• Porosity, ϕ
Chemical (7 descriptors)	• Carbon percentage, C%<br>• Hydrogen percentage, H%<br>• Nitrogen percentage, N%<br>• Oxygen percentage, O%<br>• Metalloid Percentage, Me%<br>• Ametal Percentage, Ametal%<br>• Halogen Percentage, Halogen%
Energy-based (1 descriptor)	• Henry's coefficients of gases (mol/kg/Pa), $K_{H,i}$

Pearson correlation coefficients (r) were calculated to evaluate the interdependencies among these 12 features and 4 target data (simulated gas uptakes at 0.1, 1, 5, 10 bar), and these correlations are presented in **Figures S1-S5** for each gas molecule of interest.

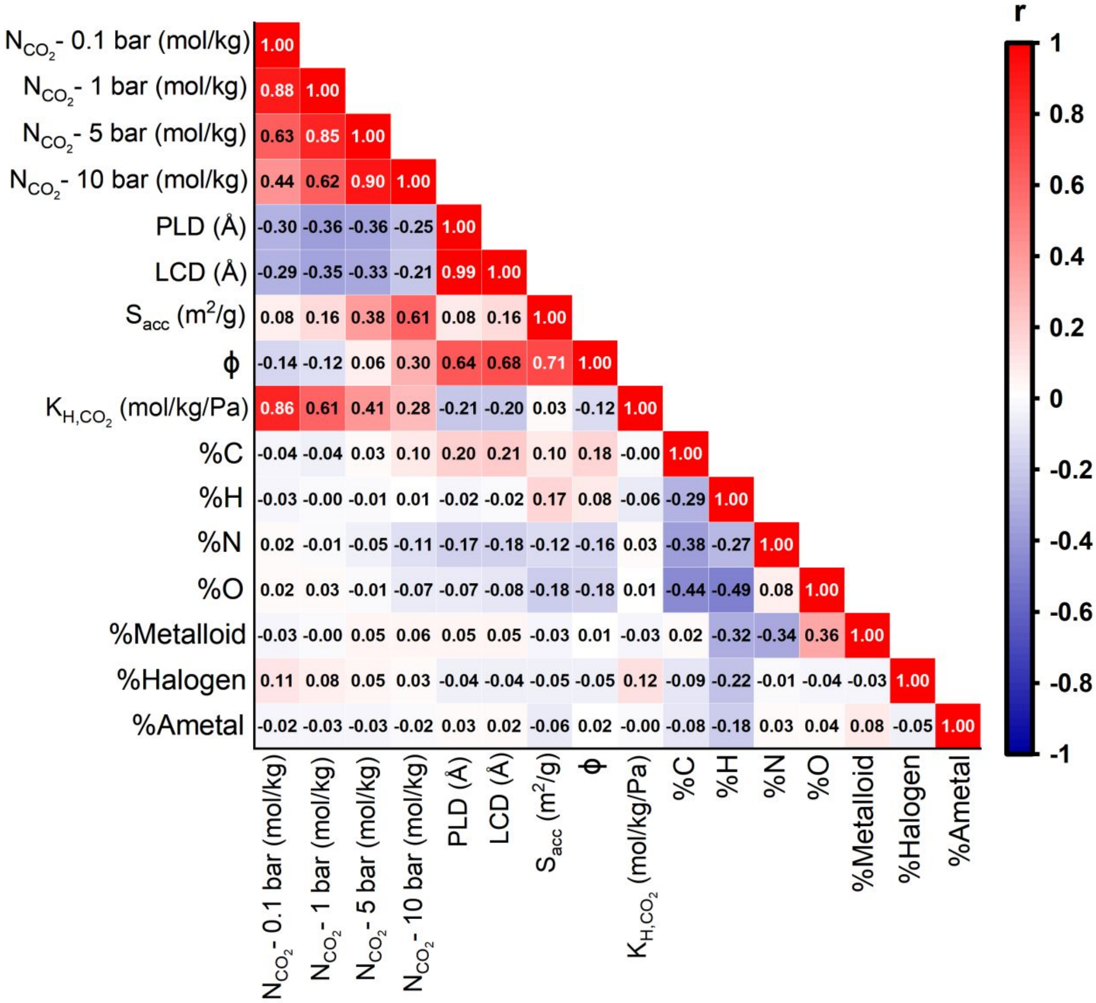

Figure S1. Correlation matrix for CO_2 uptakes, and structural, chemical, energetic descriptors of 1,060 CoRE COFs. Pearson coefficients (r) are provided for the relationship between each descriptor.

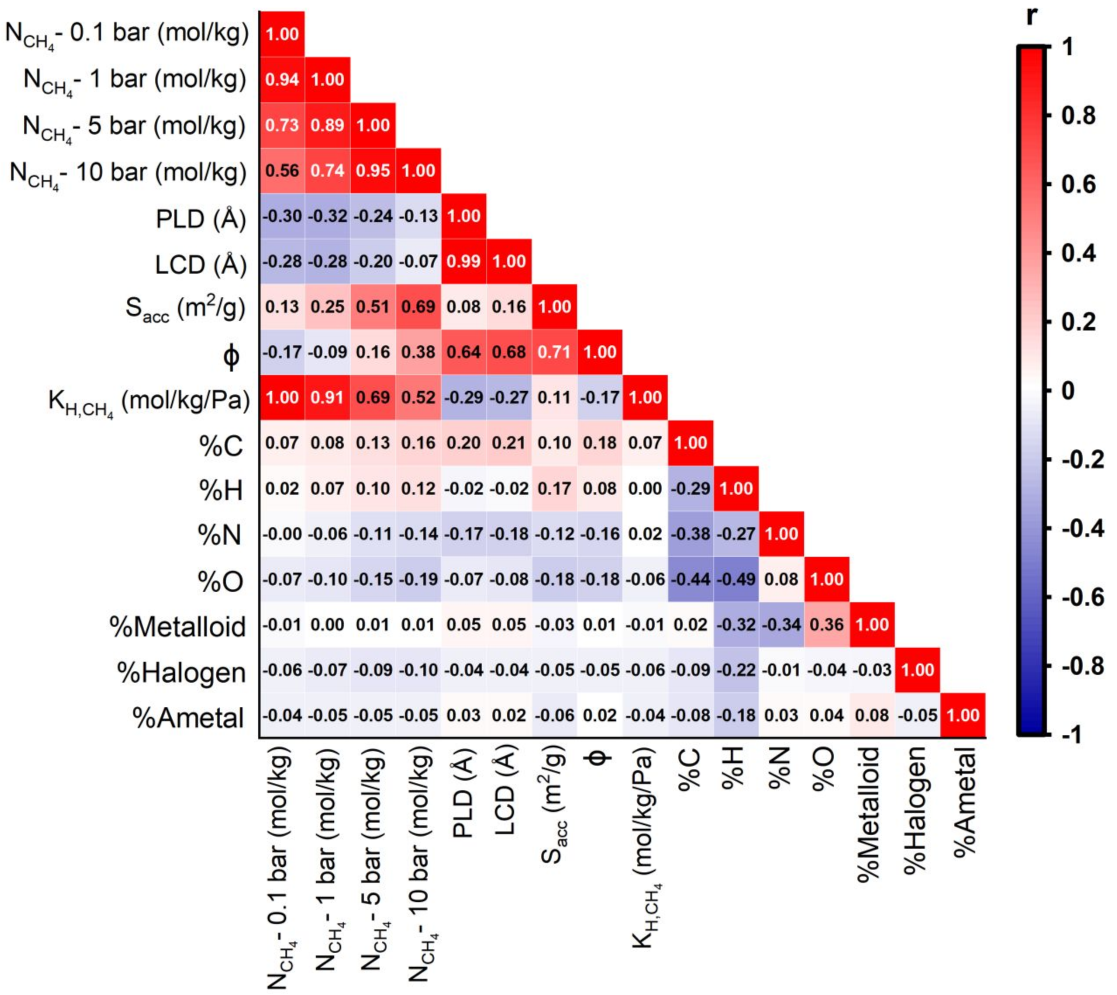

Figure S2. Correlation matrix for CH_4 uptakes, and structural, chemical, energetic descriptors of 1,060 CoRE COFs. Pearson coefficients (r) are provided for the relationship between each descriptor.

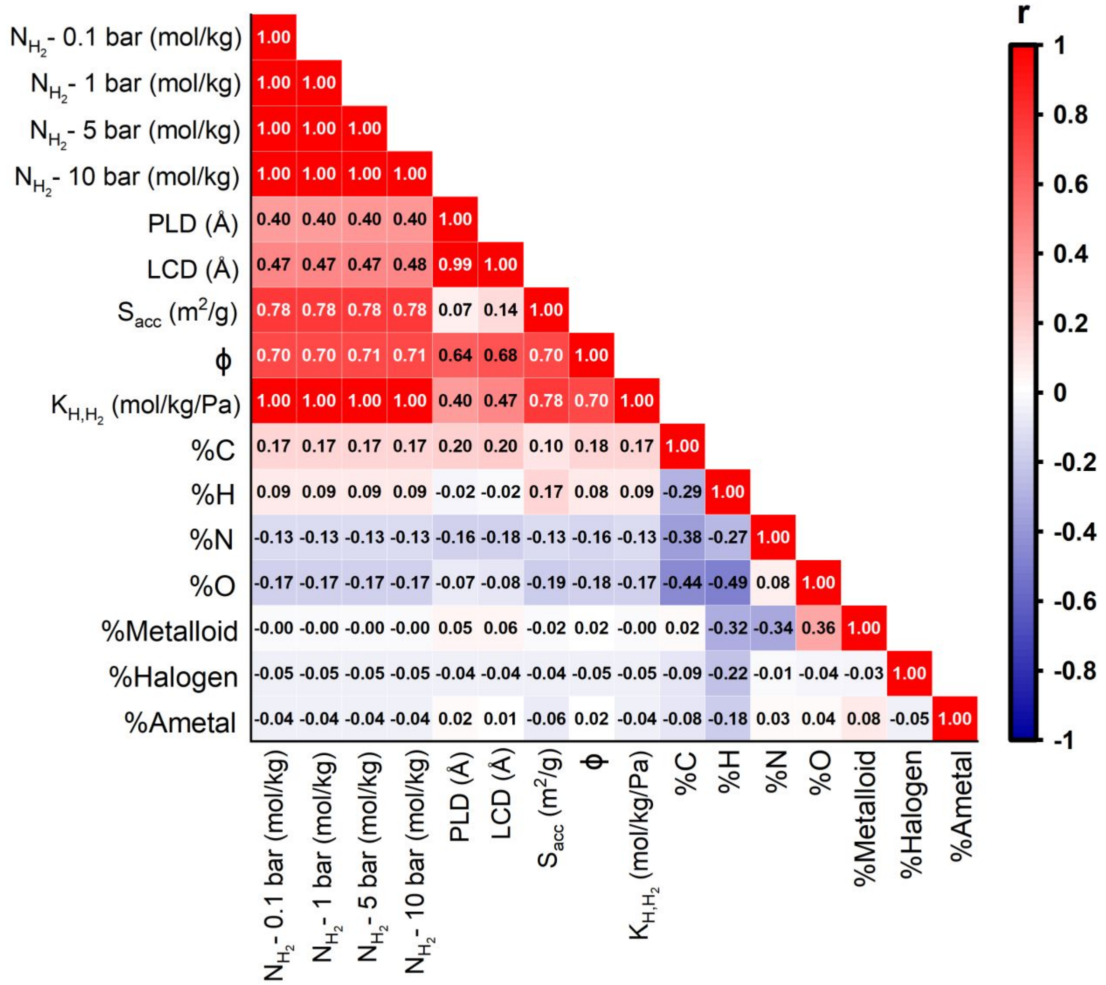

Figure S3. Correlation matrix for H_2 uptakes, and structural, chemical, energetic descriptors of 1,060 CoRE COFs. Pearson coefficients (r) are provided for the relationship between each descriptor.

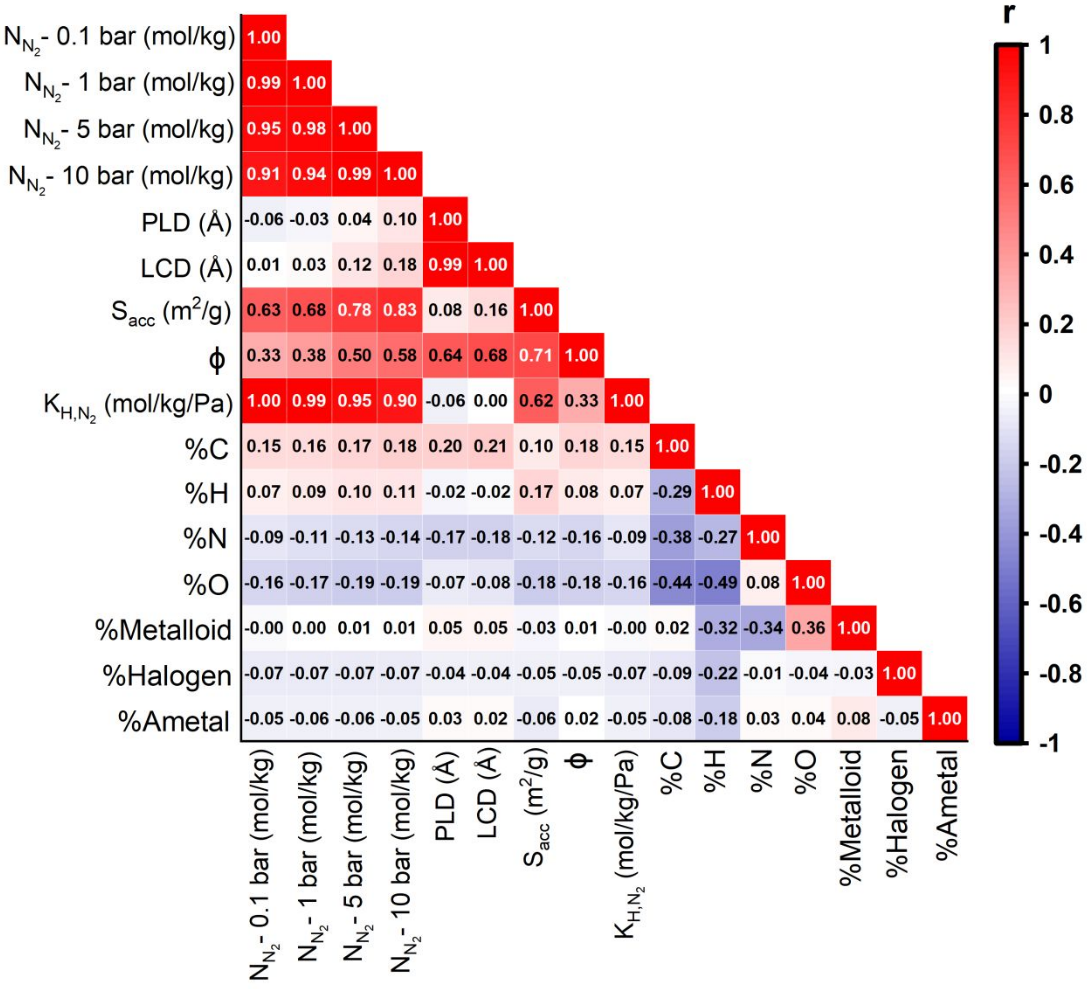

Figure S4. Correlation matrix for N_2 uptakes, and structural, chemical, energetic descriptors of 1,060 CoRE COFs. Pearson coefficients (r) are provided for the relationship between each descriptor.

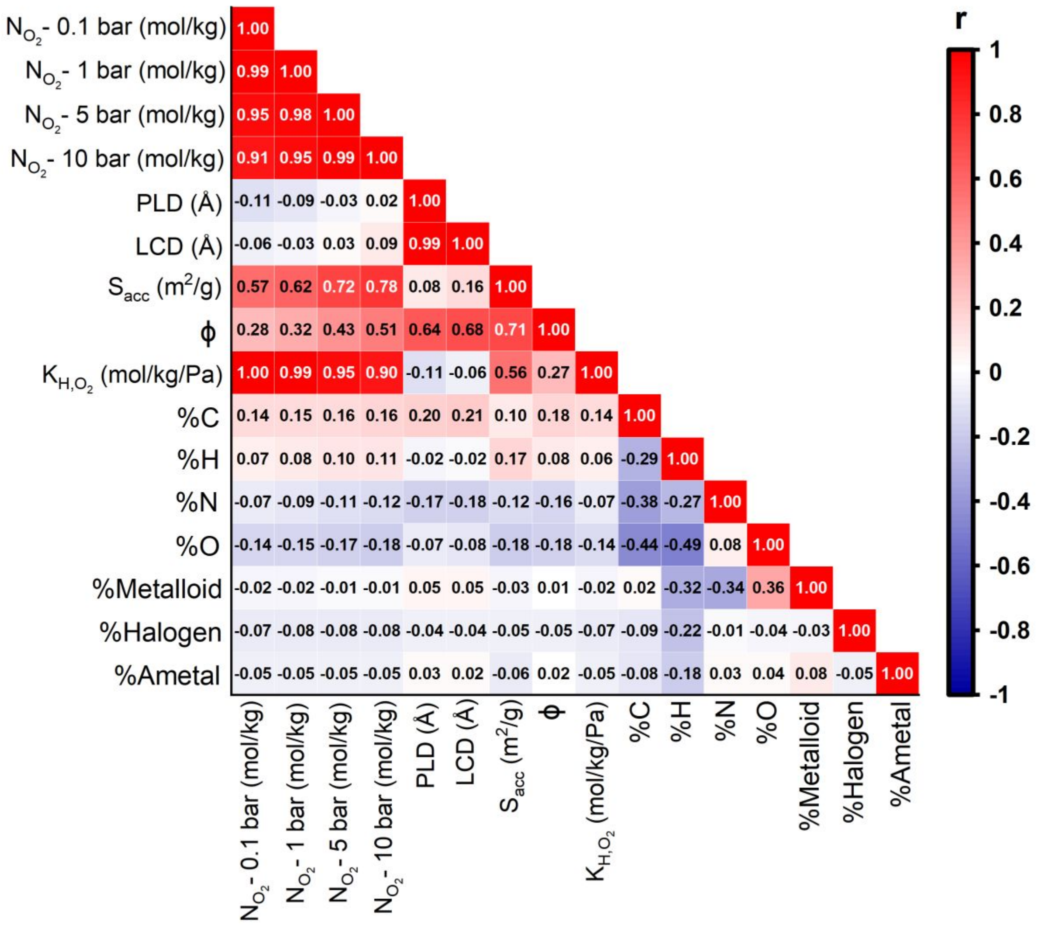

Figure S5. Correlation matrix for O_2 uptakes, and structural, chemical, energetic descriptors of 1,060 CoRE COFs. Pearson coefficients (r) are provided for the relationship between each descriptor.

We used tree-based pipeline optimization tool (TPOT)[19] within the automated machine learning (AutoML) framework to identify the most appropriate ML algorithms and optimize their hyperparameters. In TPOT, regression algorithms from the scikit-learn[20] library were employed for model selection. A stratified sampling approach was adopted to ensure a consistent distribution of features across the training and test datasets, with 80% of the data allocated for training and 20% for testing. To prevent overfitting, 5-fold cross-validation was applied. The performance of the developed ML models was evaluated using various statistical accuracy metrics, including the coefficient of determination (R^2), mean absolute error (MAE), root mean square error (RMSE), and Spearman's rank correlation coefficient (SRCC), as explained in **Table S2**. Based on these metrics, different regression models such as Extra Trees,[21] GradientBoost,[22] XG-Boost,[23] ElasticNet,[24] and RidgeCV[25] were selected depending on their accuracy in predicting target gas uptakes at the corresponding conditions as listed in detail in **Tables S3-S6**.

Table S2. The statistical accuracy metrics calculated to evaluate ML models.

**Metric**	**Formula**
**Pearson Correlation Constant (r)**	$\frac{\sum_{i=1}^{n}(x_i - \bar{x})(y_i - \bar{y})}{\sqrt{\sum_{i=1}^{n}(x_i - \bar{x})^2}\sqrt{\sum_{i=1}^{n}(y_i - \bar{y})^2}}$
**Coefficient of Determination (R^2)**	$1-\frac{1}{M}\sum_{m=1}^{M}(\bar{y}-\hat{y})^2 \Big/ \frac{1}{M}\sum_{m=1}^{M}(y-\hat{y})^2$
**Mean Absolute Error (MAE)**	$\sum_{m=1}^{M}\lvert y-\hat{y}\rvert / M$
**Root Mean Square Error (RMSE)**	$\sqrt{\sum_{m=1}^{M}(y-\hat{y})^2 / M}$
**Spearman Ranking Correlation Coefficient (SRCC)**	$1-\frac{6\sum d_i^2}{M(M^2-1)}$

M: the number of samples, x_i: i^{th} data point of variable 1, y_i: i^{th} data point of variable 2, y: simulated value, $\hat{y}$: predicted value, $\bar{y}$: average of the simulated value, d_i: the difference between the ranks of corresponding variables.

Table S3. The ML pipelines and their parameters based on each target gas adsorption properties of 1,060 CoRE COFs at 0.1 bar, 298 K.

Property	Best Pipeline with Parameters
CO_2	ExtraTreesRegressor(input_matrix,bootstrap=False,max_features=0.7500000000000001, min_samples_leaf=1,min_samples_split=4, n_estimators=100)
CH_4	RidgeCV(PolynomialFeatures(RobustScaler(input_matrix),degree=2,include_bias=False, interaction_only=False))
H_2	ElasticNetCV(RobustScaler(input_matrix), l1_ratio=0.65, tol=0.001)
N_2	ElasticNetCV(SelectPercentile(MinMaxScaler(input_matrix),percentile=41),l1_ratio=0.45, tol=0.001)
O_2	ElasticNetCV(MaxAbsScaler(input_matrix), l1_ratio=0.8, tol=0.0001)

Table S4. The ML pipelines and their parameters based on each target gas adsorption properties of 1,060 CoRE COFs at 1 bar, 298 K.

Property	Best Pipeline with Parameters
CO_2	XGBRegressor(input_matrix, learning_rate=0.1, max_depth=3, min_child_weight=7, n_estimators=100, n_jobs=1, objective=reg:squarederror, subsample=0.4, verbosity=0)
CH_4	XGBRegressor(XGBRegressor(input_matrix, colsample_bytree=1.0, learning_rate=0.1, max_depth=4,n_estimators=100,subsample=1.0),colsample_bytree=1.0,learning_rate=0.1, max_depth=8, n_estimators=100, subsample=0.7)
H_2	RidgeCV(RobustScaler(MaxAbsScaler(input_matrix)))
N_2	RidgeCV(RidgeCV(StandardScaler(PolynomialFeatures(input_matrix, degree=2, include_bias=False, interaction_only=False))))
O_2	RidgeCV(StandardScaler(PolynomialFeatures(input_matrix, degree=2, include_bias=False, interaction_only=False)))

Table S5. The ML pipelines and their parameters based on each target gas adsorption properties of 1,060 CoRE COFs at 5 bar, 298 K.

Property	Best Pipeline with Parameters
CO_2	XGBRegressor(input_matrix, learning_rate=0.1, max_depth=6, min_child_weight=9, n_estimators=100, n_jobs=1, objective=reg:squarederror, subsample=0.8500000000000001, verbosity=0)
CH_4	XGBRegressor(input_matrix, learning_rate=0.1, max_depth=4, min_child_weight=5, n_estimators=100,n_jobs=1,objective=reg:squarederror,subsample=0.8500000000000001, verbosity=0)
H_2	RidgeCV(DecisionTreeRegressor(StandardScaler(input_matrix), max_depth=10, min_samples_leaf=1, min_samples_split=19))
N_2	ExtraTreesRegressor(input_matrix,bootstrap=False,max_features=0.7000000000000001, min_samples_leaf=1, min_samples_split=2, n_estimators=100)
O_2	XGBRegressor(RidgeCV(input_matrix), learning_rate=0.1, max_depth=3, min_child_weight=1, n_estimators=100, n_jobs=1, objective=reg:squarederror, subsample=0.45, verbosity=0)

Table S6. The ML pipelines and their parameters based on each target gas adsorption properties of 1,060 CoRE COFs at 10 bar, 298 K.

Property	Best Pipeline with Parameters
CO_2	XGBRegressor(input_matrix, learning_rate=0.1, max_depth=4, min_child_weight=5, n_estimators=100,n_jobs=1,objective=reg:squarederror,subsample=0.8500000000000001, verbosity=0)
CH_4	XGBRegressor(input_matrix, learning_rate=0.1, max_depth=5, min_child_weight=2, n_estimators=100, n_jobs=1, objective=reg:squarederror, subsample=0.5, verbosity=0)
H_2	RidgeCV(StandardScaler(input_matrix))
N_2	ExtraTreesRegressor(MinMaxScaler(input_matrix),bootstrap=False,max_features=0.6500000000000001, min_samples_leaf=1, min_samples_split=2, n_estimators=100)
O_2	ExtraTreesRegressor(MaxAbsScaler(input_matrix),bootstrap=False,max_features=0.8500000000000001, min_samples_leaf=1, min_samples_split=4, n_estimators=100)

3. Development and transferability test of CoRE ML Models:

The accuracies of models were assessed by comparing ML-predicted gas uptakes with the simulated ones as shown in **Figure S6-S9** and the accuracy metrics are given in **Table S7**.

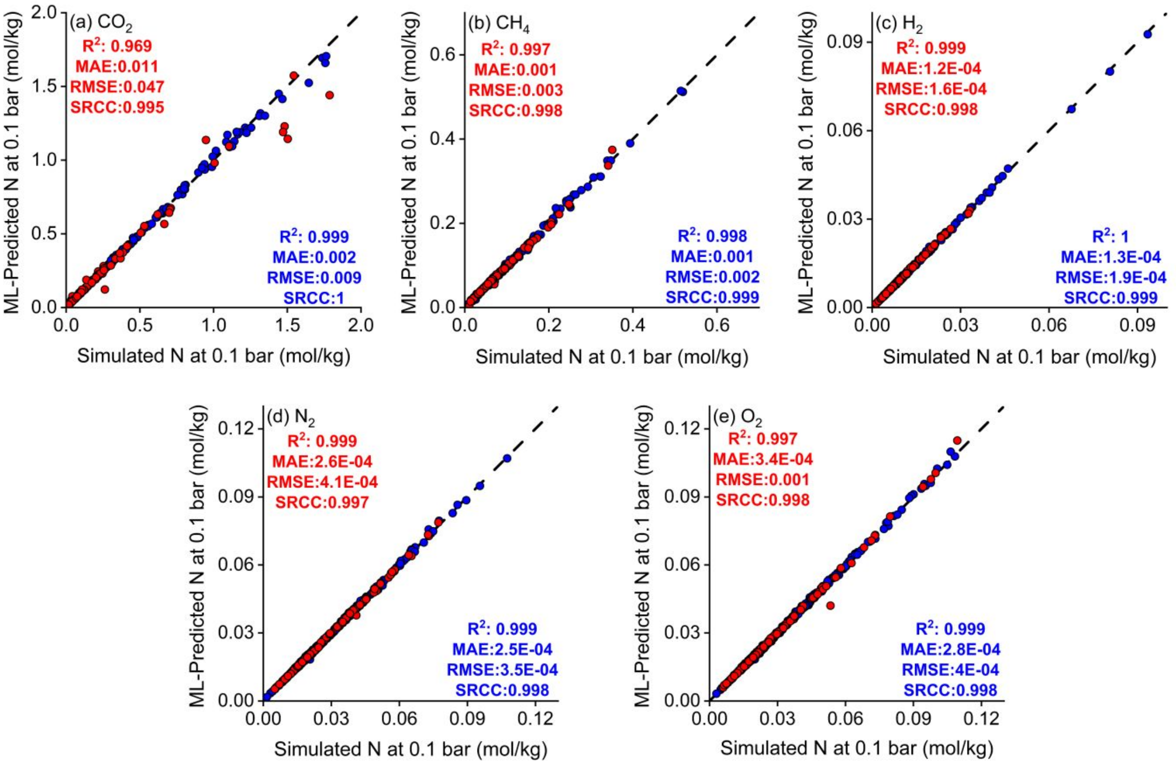

Figure S6. Comparison of ML-predicted and simulated (a) CO_2, (b) CH_4, (c) H_2, (d) N_2, (e) O_2 uptakes of 848 CoRE COFs in the training set and 212 CoRE COFs in the test set at 0.1 bar, 298 K. Blue (red) symbols represent training (test) data.

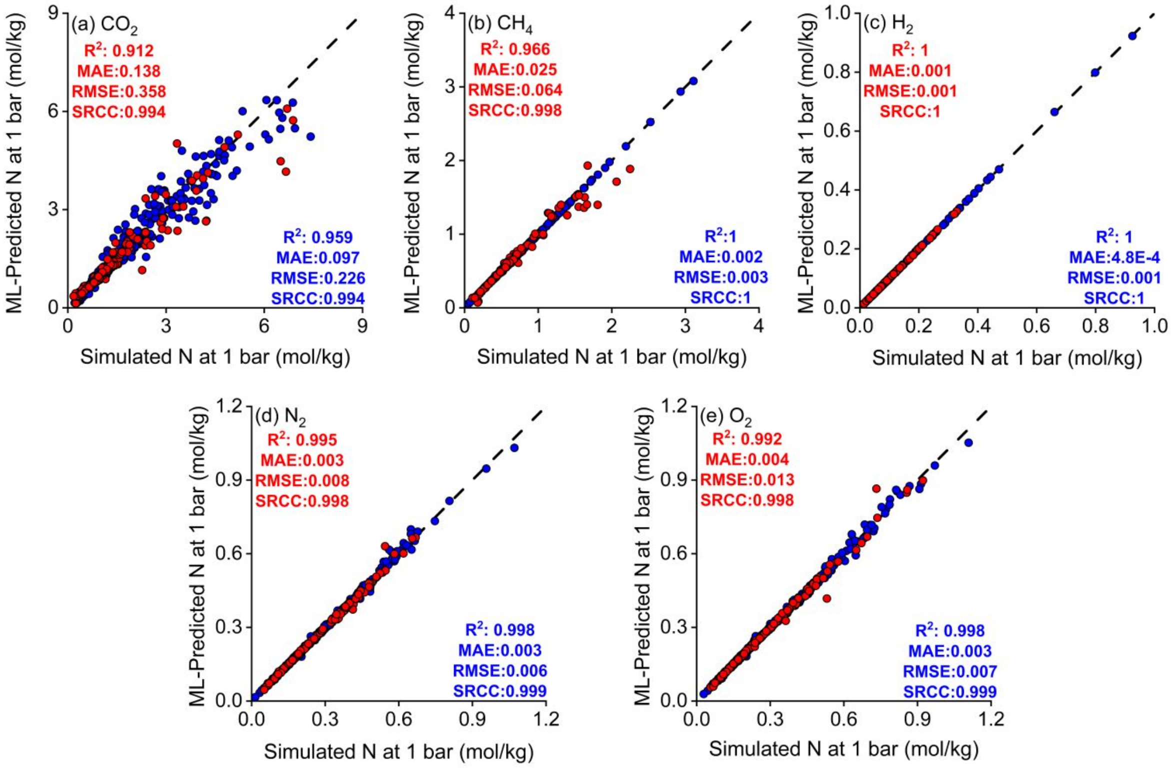

Figure S7. Comparison of ML-predicted and simulated (a) CO_2, (b) CH_4, (c) H_2, (d) N_2, (e) O_2 uptakes of 848 CoRE COFs in the training set and 212 CoRE COFs in the test set at 1 bar, 298 K. Blue (red) symbols represent training (test) data.

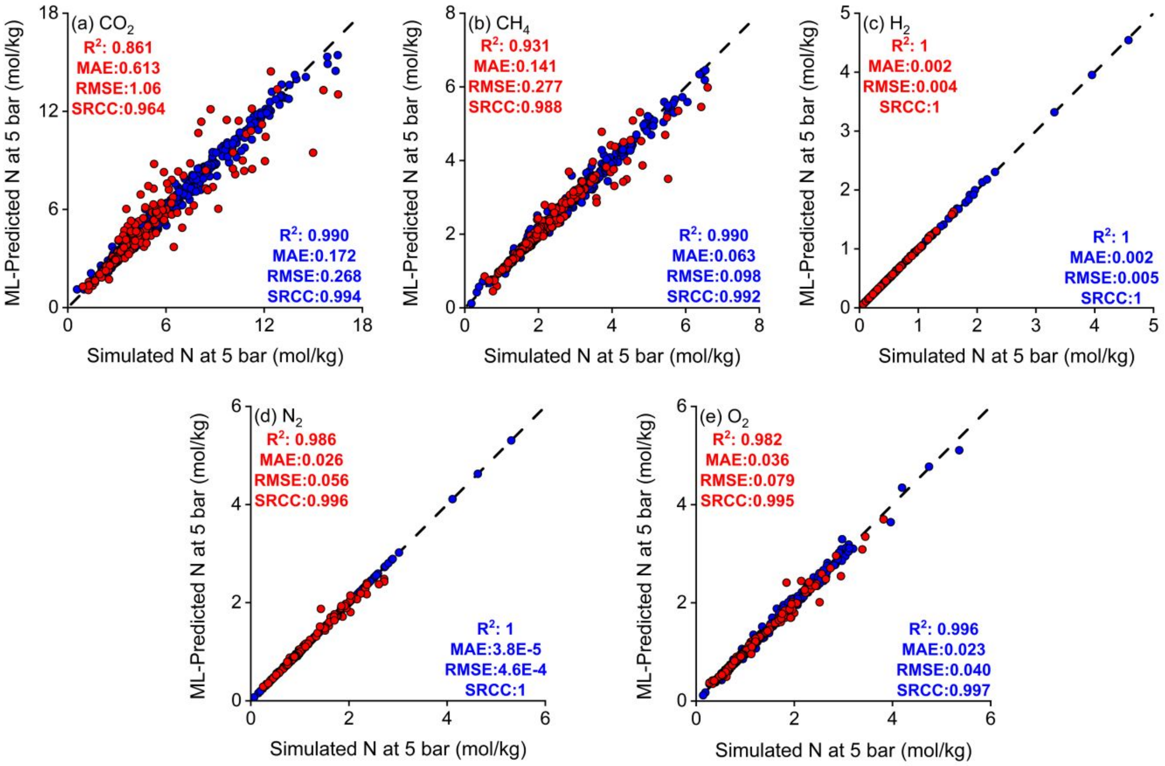

Figure S8. Comparison of ML-predicted and simulated (a) CO_2, (b) CH_4, (c) H_2, (d) N_2, (e) O_2 uptakes of 848 CoRE COFs in the training set and 212 CoRE COFs in the test set at 5 bar, 298 K. Blue (red) symbols represent training (test) data.

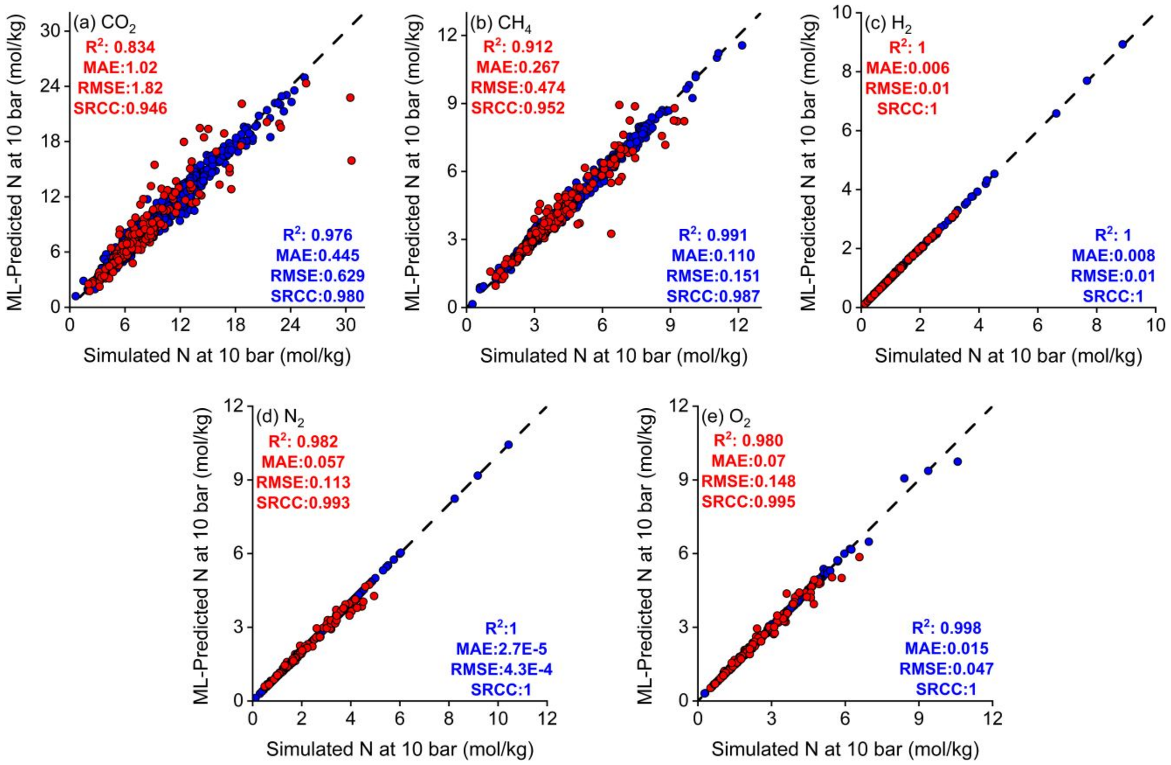

Figure S9. Comparison of ML-predicted and simulated (a) CO_2, (b) CH_4, (c) H_2, (d) N_2, (e) O_2 uptakes of 848 CoRE COFs in the training set and 212 CoRE COFs in the test set at 10 bar, 298 K. Blue (red) symbols represent training (test) data.

To test the transferability of our CoRE ML models, it was necessary to apply them to an entirely unseen hypoCOF dataset. We first identified a representative subset of 6,872 hypoCOFs, 10% of the full dataset, selected to closely match the overall hypoCOF property distributions (surface area, porosity, and $K_{H,i}$) as shown in **Figure S10**. The results showed that the selected hypoCOF subset successfully reflects the entire hypoCOF space.

Table S7. The statistical accuracy metrics calculated for CoRE ML models. R^2 and SRCC are dimensionless values ranging from 0 to 1, MAE and RMSE are expressed in units of mol/kg.

Target Data	Training Set	Test Set
CO_2-0.1 bar	R^2:0.999, MAE:0.002, RMSE:0.009, SRCC:1	R^2:0.969, MAE:0.011, RMSE:0.047, SRCC:0.995
CO_2-1 bar	R^2:0.959, MAE:0.097, RMSE:0.226, SRCC:0.994	R^2:0.912, MAE:0.138, RMSE:0.358, SRCC:0.994
CO_2-5 bar	R^2:0.990, MAE:0.172, RMSE:0.268, SRCC:0.994	R^2:0.861, MAE:0.613, RMSE:1.06, SRCC:0.964
CO_2-10 bar	R^2:0.976, MAE:0.445, RMSE:0.629, SRCC:0.980	R^2:0.834, MAE:1.02, RMSE:1.82, SRCC:0.946
CH_4-0.1 bar	R^2:0.998, MAE:0.001, RMSE:0.002, SRCC:0.999	R^2:0.997, MAE:0.001, RMSE:0.003, SRCC:0.998
CH_4-1 bar	R^2:1,MAE:0.002, RMSE:0.003, SRCC:1	R^2:0.966, MAE:0.025, RMSE:0.064, SRCC:0.998
CH_4-5 bar	R^2:0.990, MAE:0.063, RMSE:0.098, SRCC:0.992	R^2:0.931, MAE:0.141, RMSE:0.277, SRCC:0.988
CH_4-10 bar	R^2:0.991, MAE:0.110, RMSE:0.151, SRCC:0.987	R^2:0.912, MAE:0.267, RMSE:0.474, SRCC:0.952
H_2-0.1 bar	R^2:0.999, MAE:1.3E-4, RMSE:1.9E-4, SRCC:0.999	R^2:0.999, MAE:1.2E-4, RMSE:1.6E-4, SRCC:0.998
H_2-1 bar	R^2:1, MAE:4.8E-4, RMSE:0.001, SRCC:1	R^2:1, MAE:0.001, RMSE:0.001, SRCC:1
H_2-5 bar	R^2:1, MAE:0.002, RMSE:0.005, SRCC:1	R^2:1, MAE:0.002, RMSE:0.004, SRCC:1
H_2-10 bar	R^2:1, MAE:0.008, RMSE:0.012, SRCC:1	R^2:1, MAE:0.006, RMSE:0.01, SRCC:1
N_2-0.1 bar	R^2:0.999, MAE:2.5E-4, RMSE:3.5E-4, SRCC:0.998	R^2:0.999, MAE: 2.6E-4, RMSE:4.1E-4, SRCC:0.997
N_2-1 bar	R^2:0.998, MAE:0.003, RMSE:0.006, SRCC:0.999	R^2:0.995, MAE: 0.003, RMSE:0.008, SRCC:0.998
N_2-5 bar	R^2:1, MAE:3.8E-5, RMSE:4.6E-4, SRCC:1	R^2:0.986, MAE: 0.026, RMSE:0.056, SRCC:0.996
N_2-10 bar	R^2:1, MAE: 2.7E-5, RMSE: 4.3E-4, SRCC:1	R^2:0.982, MAE: 0.057, RMSE:0.113, SRCC:0.993
O_2-0.1 bar	R^2:0.999, MAE:2.8E-4, RMSE:4E-4, SRCC:0.998	R^2:0.997, MAE:3.4E-4, RMSE:0.001, SRCC:0.998
O_2-1 bar	R^2:0.998, MAE:0.003, RMSE:0.007, SRCC:0.999	R^2:0.992, MAE:0.004, RMSE:0.013, SRCC:0.998
O_2-5 bar	R^2:0.996, MAE:0.023, RMSE:0.040, SRCC:0.997	R^2:0.982, MAE:0.036, RMSE:0.079, SRCC:0.995
O_2-10 bar	R^2:0.998, MAE:0.015, RMSE:0.047, SRCC:1	R^2:0.980, MAE:0.07, RMSE:0.148, SRCC:0.995

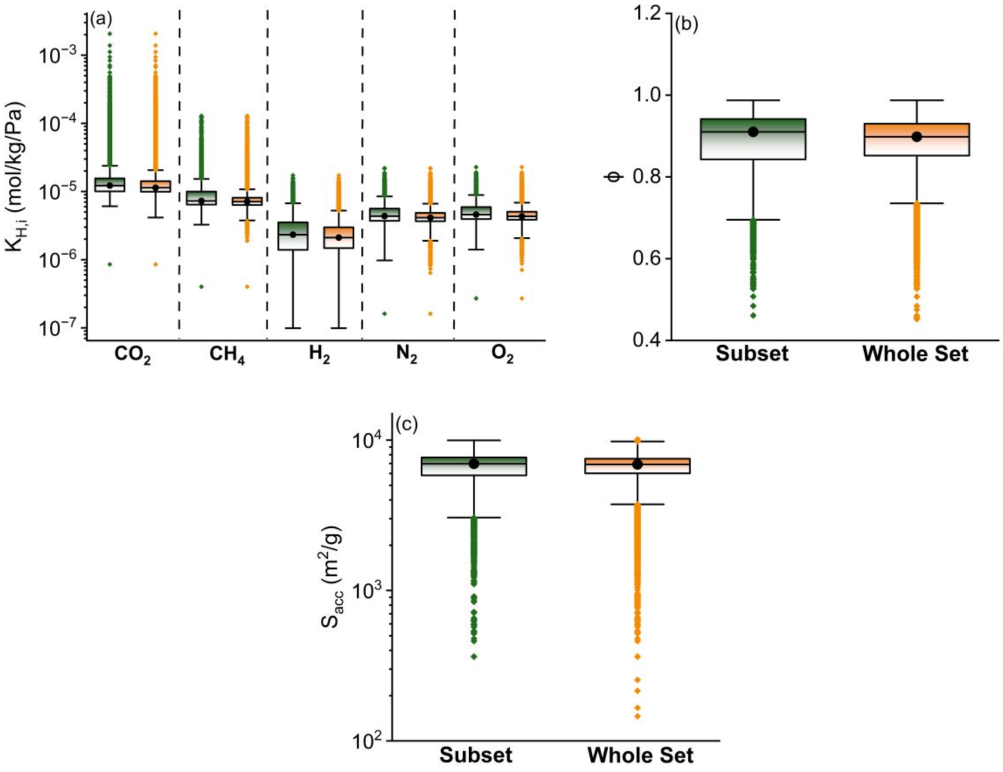

Figure S10. The box plot data distributions of hypoCOF subset comprising of 6,872 materials (dark green boxes) and whole hypoCOF material space comprising of 68,724 materials (orange boxes) with respect to their (a) Henry's constants, (b) porosities, and (c) surface areas. Points above and below the boxes represent the outliers in corresponding datasets. Black circles represent the median values of each variable in the corresponding dataset.

We then evaluated the transferability of our ML models to a distinct subset of 6,872 hypoCOFs to assess their CO_2, CH_4, H_2, N_2, and O_2 uptakes under the same conditions. **Figure S11-S14** shows the comparison between ML-predicted and simulated gas uptakes of 6,872 unseen hypoCOFs at 0.1, 1, 5 and 10 bar. The statistical accuracy metrics are given in **Table S8**. In **Figure S12(a)**, simulated CO_2 uptakes of hypoCOFs were computed in between 0.08-14 mol/kg, while ML-predicted values were -0.07-6.35 mol/kg, indicating that the CoRE ML model fails to predict CO_2 uptakes >5.5 mol/kg. This resulted in a low R^2 value, 0.585. A similar trend was observed in **Figure S12(b)**, where simulated CH_4 uptakes (0.04-7.61 mol/kg) show nearly three-fold variation compared to ML-predicted uptakes (0.07-2.72 mol/kg), yielding a very low R^2 of 0.467. In contrast to CO_2 and CH_4, ML-predicted H_2, N_2 and O_2 uptakes, 0.01-1.73, 0.02-1.63, and 0.03-1.66 mol/kg, respectively, closely aligned with the simulated values, 0.01-1.74, 0.02-1.91, and 0.03-2.04 mol/kg, in **Figures S12(c-e)**. Overall, these results showed that predicting CO_2 and CH_4 adsorption properties of hypoCOFs by directly using CoRE ML models would not be an accurate strategy and CoRE ML models should be further improved to make accurate predictions for the hypoCOF space. To achieve this, we focused on the two reasons that caused the prediction failure and proposed solutions for them.

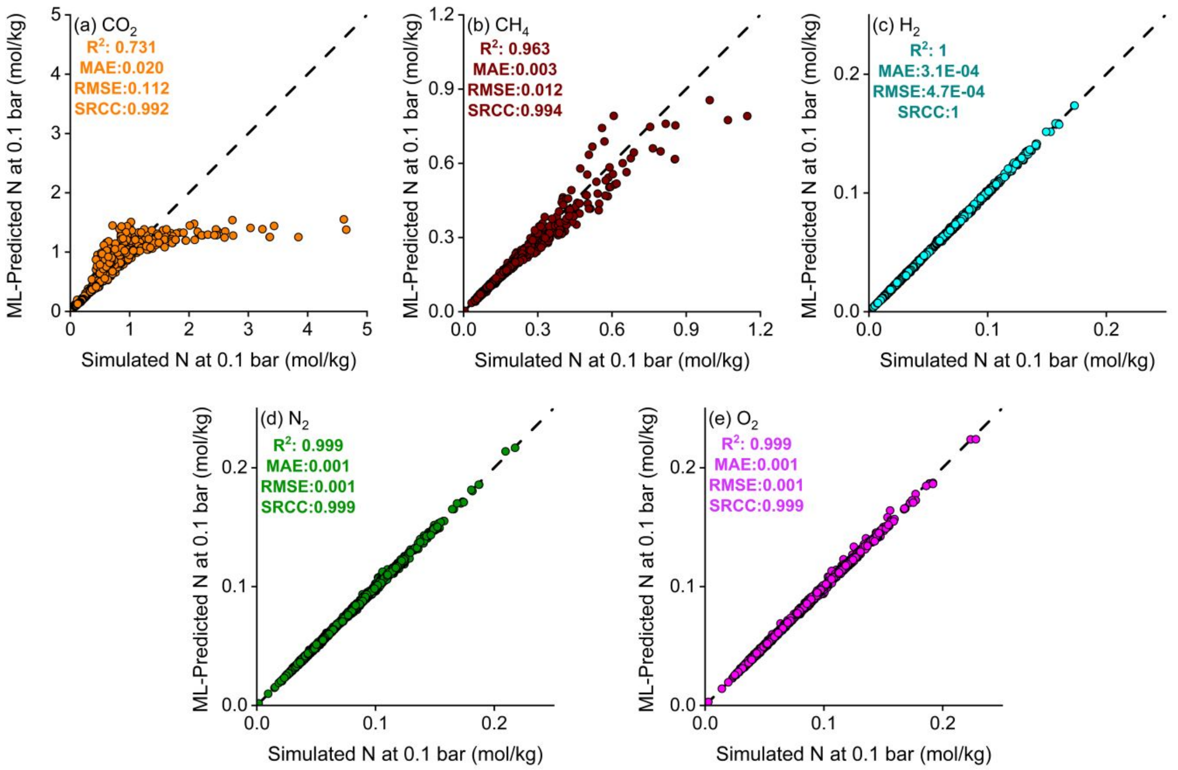

Figure S11. Comparison of ML-predicted and simulated (a) CO_2, (b) CH_4, (c) H_2, (d) N_2, (e) O_2 uptakes of 6,872 unseen hypoCOFs at 0.1 bar, 298 K.

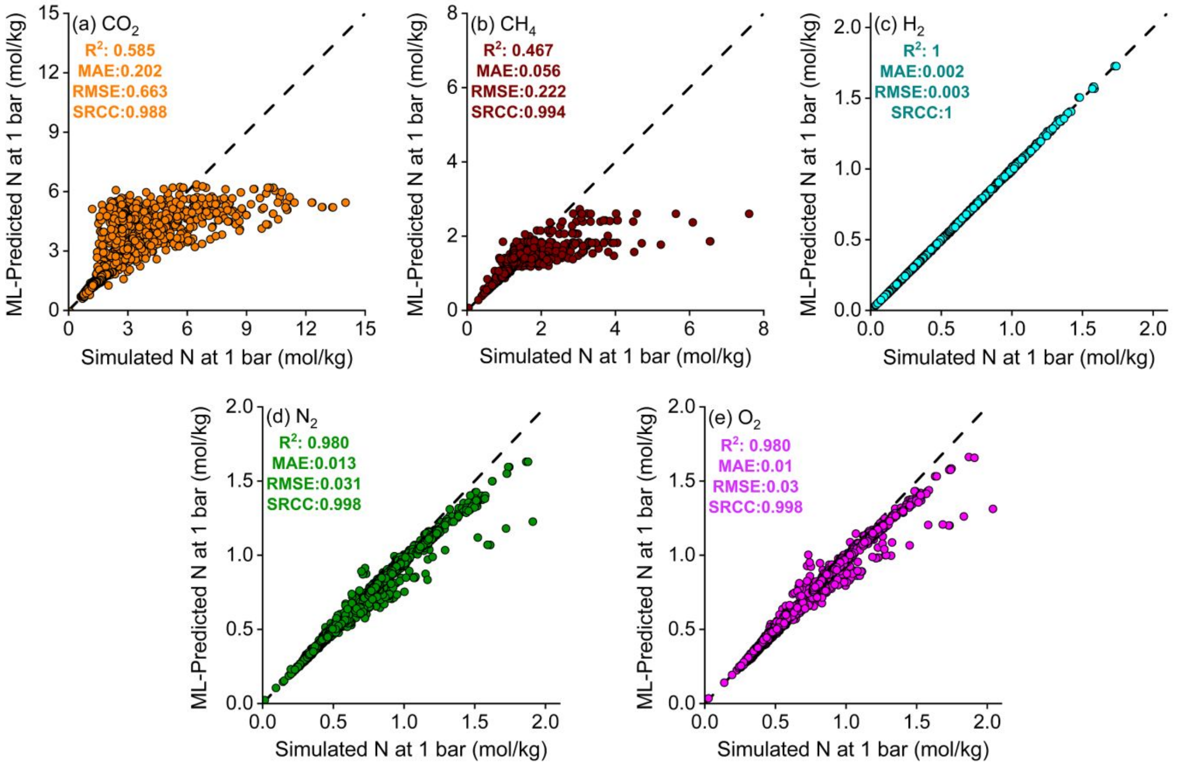

Figure S12. Comparison of ML-predicted and simulated (a) CO_2, (b) CH_4, (c) H_2, (d) N_2, (e) O_2 uptakes of 6,872 unseen hypoCOFs at 1 bar, 298 K.

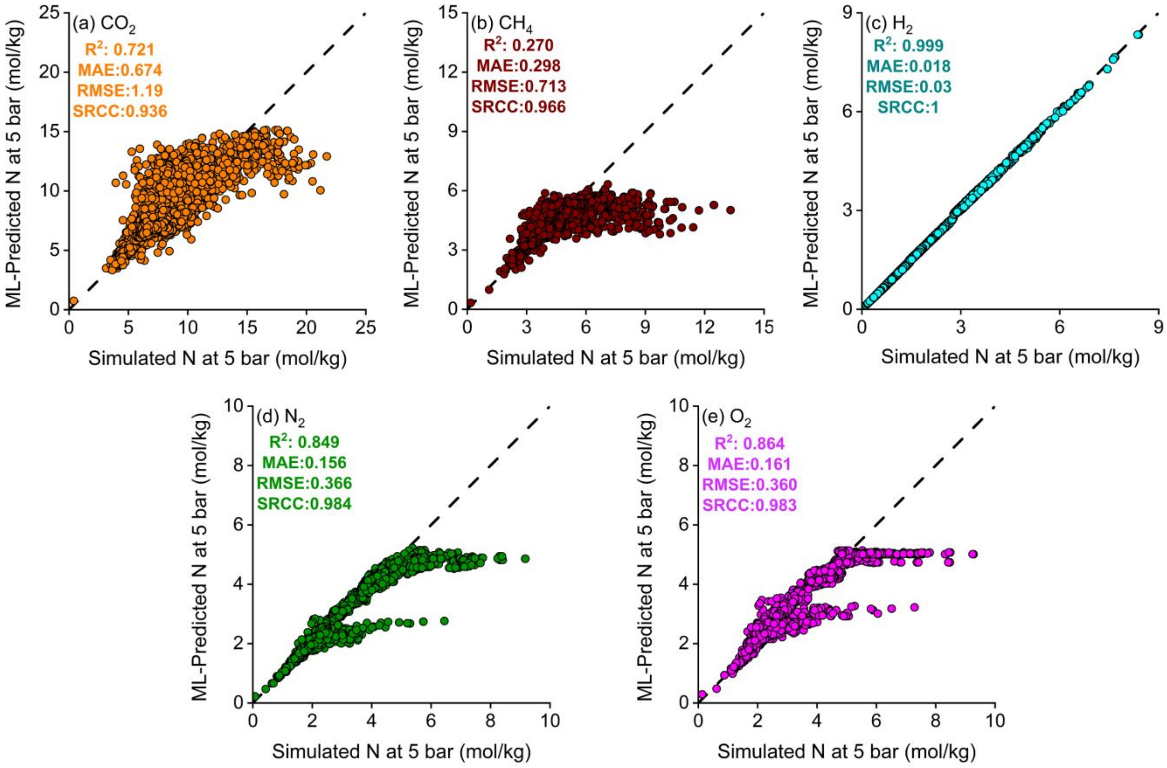

Figure S13. Comparison of ML-predicted and simulated (a) CO_2, (b) CH_4, (c) H_2, (d) N_2, (e) O_2 uptakes of 6,872 unseen hypoCOFs at 5 bar, 298 K.

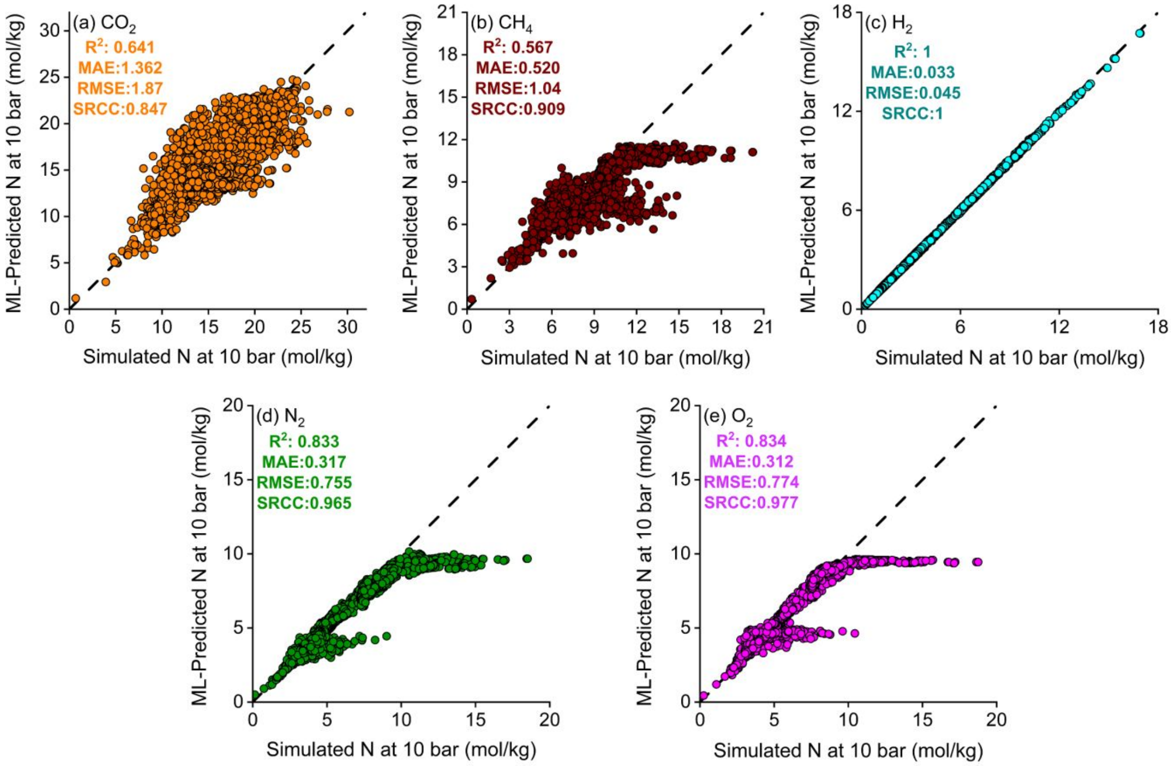

Figure S14. Comparison of ML-predicted and simulated (a) CO_2, (b) CH_4, (c) H_2, (d) N_2, (e) O_2 uptakes of 6,872 unseen hypoCOFs at 10 bar, 298 K.

Table S8. The statistical accuracy metrics calculated for the CoRE ML-predicted gas uptakes of 6,872 unseen hypoCOFs. R^2 and SRCC are dimensionless values ranging from 0 to 1, whereas MAE and RMSE are expressed in units of mol/kg.

**Target Data**	**Unseen Set**
CO_2-0.1 bar	R^2:0.731, MAE:0.020, RMSE:0.112, SRCC:0.992
CO_2-1 bar	R^2:0.585, MAE:0.202, RMSE:0.663, SRCC:0.988
CO_2-5 bar	R^2:0.721, MAE:0.674, RMSE:1.190, SRCC:0.936
CO_2-10 bar	R^2:0.641, MAE:1.362, RMSE:1.870, SRCC:0.847
CH_4-0.1 bar	R^2:0.963, MAE:0.003, RMSE:0.012, SRCC:0.994
CH_4-1 bar	R^2:0.467, MAE:0.056, RMSE:0.222, SRCC:0.994
CH_4-5 bar	R^2:0.270, MAE:0.298, RMSE:0.713, SRCC:0.966
CH_4-10 bar	R^2:0.567, MAE:0.520, RMSE:1.042, SRCC:0.909
H_2-0.1 bar	R^2:1, MAE:3.1E-04, RMSE:4.7E-4, SRCC:1
H_2-1 bar	R^2:1, MAE:0.002, RMSE:0.003, SRCC:1
H_2-5 bar	R^2:0.999, MAE:0.018, RMSE:0.03, SRCC:1
H_2-10 bar	R^2:1, MAE:0.03, RMSE:0.05, SRCC:1
N_2-0.1 bar	R^2:0.999, MAE:0.001, RMSE:0.001, SRCC:0.999
N_2-1 bar	R^2:0.980, MAE:0.013, RMSE:0.031, SRCC:0.998
N_2-5 bar	R^2:0.849, MAE:0.156, RMSE:0.366, SRCC:0.984
N_2-10 bar	R^2:0.833, MAE:0.317, RMSE:0.755, SRCC:0.965
O_2-0.1 bar	R^2:0.999, MAE:0.001, RMSE:0.001, SRCC:0.999
O_2-1 bar	R^2:0.980, MAE:0.012, RMSE:0.031, SRCC:0.998
O_2-5 bar	R^2:0.864, MAE:0.161, RMSE:0.360, SRCC:0.983
O_2-10 bar	R^2:0.834, MAE:0.312, RMSE:0.774, SRCC:0.977

Data extrapolation problem: Previous studies showed that regression models have limitations in accurately predicting data points that lie outside their training set.[26, 27] In other words, models cannot make predictions beyond what they learned. **Figure S15** compares the trained gas uptake data of CoRE ML models and simulated data of hypoCOFs that ML models attempted to predict. **Figure S15(a)** shows that gas uptakes for unseen hypothetical COFs vary widely, sometimes exceeding or falling below the ranges observed for the trained CoRE COFs for different gases. For instance, the CO_2 (CH_4) uptakes of CoRE COFs fall within 0.18-7.41 mol/kg (0.05-3.11 mol/kg), while uptakes for hypoCOFs can be significantly larger 0.08-14

mol/kg (0.04-7.61 mol/kg) at 1 bar. This implies that CoRE ML models cannot generalize the gas uptake patterns to accurately predict the adsorption data of unseen hypoCOFs. The tree-based algorithms that we used in developing the CoRE ML models for CO_2 (XGBoost), and CH_4 (XGBoost), given in **Table S4**, lack the ability to extrapolate beyond the training data, thus making inaccurate predictions for these gases. Conversely, for H_2, N_2, and O_2 adsorption data, the CoRE ML models exhibited strong extrapolative capabilities despite the notable differences between the training datasets (5×10^{-3}-0.93 mol/kg for H_2, 0.02-1.07 mol/kg for N_2 and 0.03-1.11 mol/kg for O_2) and the unseen datasets (0.01-1.74 mol/kg for H_2, 0.02-1.91 mol/kg for N_2, and 0.03-2.04 mol/kg for O_2). We attributed this to the use of linear ML models based on RidgeCV algorithm, which can perform linear regression that effectively extrapolates beyond the training set, especially when multiple features are closely aligned with the target variable, as shown by the correlation coefficients between H_2, N_2 and O_2 uptakes and the features in **Figures S3-S5**.

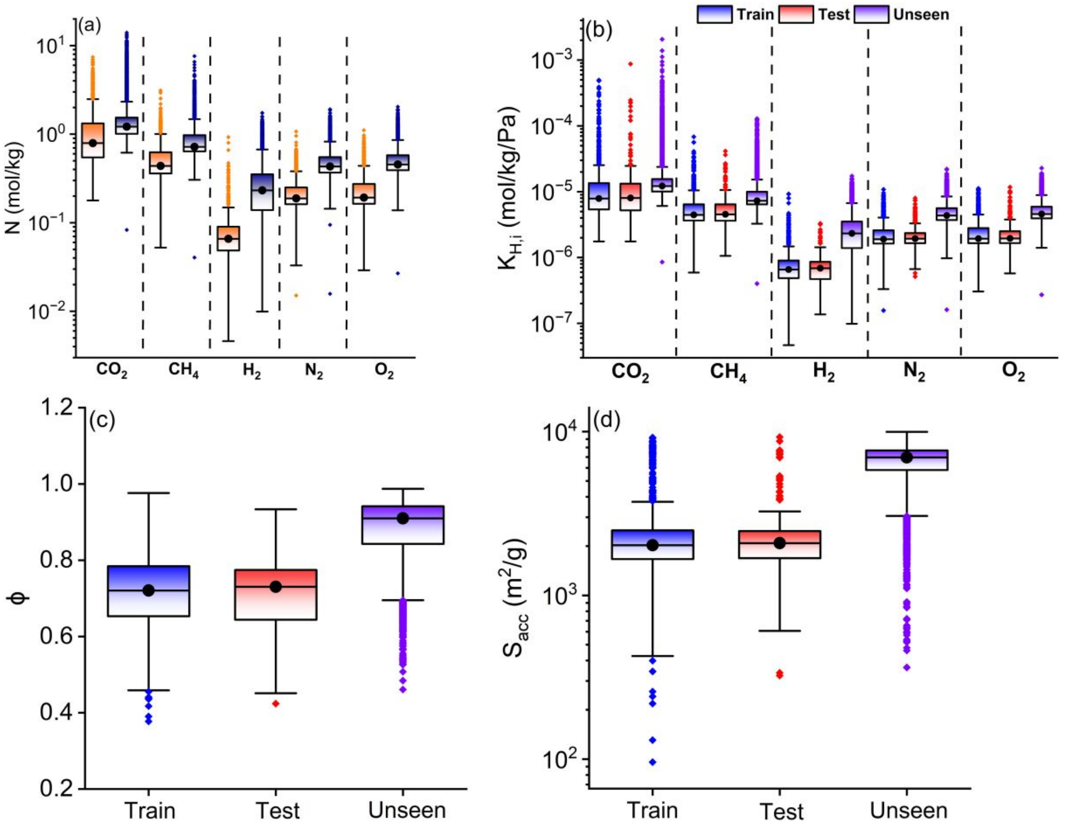

Figure S15. (a) Simulated gas uptake data of 848 CoRE COFs used in training CoRE ML models (orange boxes) and simulated gas uptake data of 6,872 unseen hypoCOFs (dark blue boxes) at 1 bar. (b) Henry's constants, (c) porosities, and (d) surface areas of 848 CoRE COFs used in training CoRE ML models (blue boxes) and 212 CoRE COFs used in testing CoRE ML models (red boxes), compared with those of 6,872 unseen hypoCOFs (violet boxes). Points above and below the boxes represent the outliers in corresponding datasets. Black circles represent the median values of each variable in the corresponding dataset.

Feature generalization problem: Another issue with CoRE ML models is that the feature ranges used in their training sets were insufficient to cover the unseen hypoCOFs. **Figures S15(b-d)** present the box plot distributions of the three most correlated features, Henry's constants, surface area, and porosity, for the training set of CoRE COFs and unseen hypoCOFs. The Henry's constants and porosities of CoRE COFs adequately generalize these features of hypoCOFs. However, there is a substantial disparity in surface area distributions: only 8% of CoRE COFs have surface areas >5,000 m^2/g compared to 86% of selected hypoCOFs in unseen dataset and 89% of the entire hypoCOF database, highlighting the significant bias that undermines the transferability of CoRE ML models.

4. Development and transferability test of CoRE+Hypo ML Models:

To address these issues, we retrained the CoRE ML models using an additional set of 563 hypoCOFs as discussed in the main manuscript. These hypoCOFs were specifically chosen to (i) exhibit gas uptakes higher than any values recorded in the original CoRE ML training set (listed in **Table S9**) and (ii) possess surface areas greater than 5,000 m^2/g. All details regarding the newly retrained models, referred to as "CoRE+Hypo ML models," are provided. **Table S10** compares the trained structural and energetic features of the CoRE and CoRE+Hypo ML models, and **Tables S11-S14** summarize all twenty CoRE+Hypo ML models along with their optimized hyperparameters.

Table S9. Comparison of trained data ranges for CoRE ML model and CoRE+Hypo ML model.

Target Data	CoRE ML	CoRE+Hypo ML
CO_2-0.1 bar (mol/kg)	0.02-1.76	0.01-4.65
CO_2-1 bar (mol/kg)	0.20-6.74	0.08-14
CO_2-5 bar (mol/kg)	0.57-16.49	0.40-21.71
CO_2-10 bar (mol/kg)	0.65-25.53	0.65-30.62
CH_4-0.1 bar (mol/kg)	0.01-0.52	4×10^{-3}-1.15
CH_4-1 bar (mol/kg)	0.05-3.11	0.04-7.61
CH_4-5 bar (mol/kg)	0.18-6.54	0.18-13.31
CH_4-10 bar (mol/kg)	0.26-12.17	0.26-20.24
H_2-0.1 bar (mol/kg)	4.8×10^{-4}-0.09	4.8×10^{-4}-0.17
H_2-1 bar (mol/kg)	5×10^{-3}-0.93	5×10^{-3}-1.73
H_2-5 bar (mol/kg)	0.02-4.57	0.02-8.40
H_2-10 bar (mol/kg)	0.05-8.88	0.05-16.93
N_2-0.1 bar (mol/kg)	2×10^{-3}-0.11	1.5×10^{-3}-0.22
N_2-1 bar (mol/kg)	0.02-1.07	0.02-1.91
N_2-5 bar (mol/kg)	0.07-5.31	0.07-9.17
N_2-10 bar (mol/kg)	0.12-10.43	0.12-18.45
O_2-0.1 bar (mol/kg)	3×10^{-3}-0.11	2.7×10^{-3}-0.23
O_2-1 bar (mol/kg)	0.03-1.11	0.03-2.04
O_2-5 bar (mol/kg)	0.14-5.36	0.13-9.28
O_2-10 bar (mol/kg)	0.28-10.58	0.25-18.64

Table S10. Comparison of trained feature data ranges for CoRE ML model and CoRE+Hypo ML model. Median values are given in the parentheses for corresponding ranges of each feature.

Features	CoRE	CoRE+Hypo
PLD (Å)	3.95-89.1 (17.4)	3.82-92.9 (18.8)
LCD (Å)	4.35-89.2 (18.3)	4.35-93 (20.0)
ϕ	0.38-0.98 (0.72)	0.38-0.99 (0.75)
S_{acc} (m^2/g)	95.82-9,197.9 (2,025.8)	95.82-9,960 (2,405.6)
K_{H,CO_2} (mol/kg/Pa)	1.8×10^{-6}-4.9×10^{-4} (7.8×10^{-6})	8.6×10^{-7}-2.1×10^{-3} (1.4×10^{-5})
K_{H,CH_4} (mol/kg/Pa)	5.9×10^{-7}-6.8×10^{-5} (4.5×10^{-6})	4×10^{-7}-1.3×10^{-4} (6.5×10^{-5})
K_{H,H_2} (mol/kg/Pa)	4.7×10^{-8}-9.2×10^{-6} (6.6×10^{-7})	4.7×10^{-8}-1.7×10^{-5} (8.3×10^{-7})
K_{H,N_2} (mol/kg/Pa)	1.6×10^{-7}-1.1×10^{-5} (1.9×10^{-6})	1.6×10^{-7}-2.2×10^{-5} (2.6×10^{-6})
K_{H,O_2} (mol/kg/Pa)	3.1×10^{-7}-1.1×10^{-5} (1.9×10^{-6})	2.7×10^{-7}-2.3×10^{-5} (2.9×10^{-6})

Table S11. The ML pipelines and their parameters based on each target gas adsorption properties of 1,060 CoRE COFs and 563 hypoCOFs at 0.1 bar, 298 K

.

Property	Best Pipeline with Parameters
CO_2	XGBRegressor(input_matrix, learning_rate=0.1, max_depth=3, min_child_weight=1, n_estimators=100,n_jobs=1,objective=reg:squarederror,subsample=0.6500000000000001, verbosity=0)
CH_4	ExtraTreesRegressor(input_matrix,bootstrap=False,max_features=0.8,min_samples_leaf=1, min_samples_split=3, n_estimators=100)
H_2	ElasticNetCV(MaxAbsScaler(input_matrix), l1_ratio=0.9, tol=1e-05)
N_2	RidgeCV(FastICA(RandomForestRegressor(MaxAbsScaler(StandardScaler(input_matrix)), bootstrap=False, max_features=0.1, min_samples_leaf=10, min_samples_split=2, n_estimators=100), tol=0.5))
O_2	RidgeCV(FastICA(MaxAbsScaler(input_matrix), tol=0.15000000000000002))

Table S12. The ML pipelines and their parameters based on each target gas adsorption properties of 1,060 CoRE COFs and 563 hypoCOFs at 1 bar, 298 K.

Property	Best Pipeline with Parameters
CO_2	ExtraTreesRegressor(input_matrix,bootstrap=False,max_features=0.9000000000000001, min_samples_leaf=1, min_samples_split=8, n_estimators=100)
CH_4	ExtraTreesRegressor(input_matrix,bootstrap=False,max_features=0.9000000000000001, min_samples_leaf=1, min_samples_split=3, n_estimators=100)
H_2	RidgeCV(RobustScaler(input_matrix))
N_2	ExtraTreesRegressor(input_matrix,bootstrap=False,max_features=0.9500000000000001, min_samples_leaf=1, min_samples_split=2, n_estimators=100)
O_2	RandomForestRegressor(RidgeCV(RobustScaler(input_matrix)), bootstrap=False, max_features=0.4, min_samples_leaf=1, min_samples_split=5, n_estimators=100)

Table S13. The ML pipelines and their parameters based on each target gas adsorption properties of 1,060 CoRE COFs and 563 hypoCOFs at 5 bar, 298 K.

Property	Best Pipeline with Parameters
CO_2	ExtraTreesRegressor(input_matrix,bootstrap=False,max_features=1.0,min_samples_leaf=1, min_samples_split=3, n_estimators=100)
CH_4	XGBRegressor(CombineDFs(input_matrix, CombineDFs(input_matrix, input_matrix)), colsample_bytree=0.7, learning_rate=0.1, max_depth=6, n_estimators=100, subsample=0.7)
H_2	RidgeCV(FastICA(PolynomialFeatures(input_matrix, degree=2, include_bias=False, interaction_only=False), tol=0.6000000000000001))
N_2	ExtraTreesRegressor(input_matrix,bootstrap=False,max_features=1.0,min_samples_leaf=2, min_samples_split=2, n_estimators=100)
O_2	ExtraTreesRegressor(input_matrix,bootstrap=False,max_features=0.8500000000000001, min_samples_leaf=2, min_samples_split=2, n_estimators=100)

Table S14. The ML pipelines and their parameters based on each target gas adsorption properties of 1,060 CoRE COFs and 563 hypoCOFs at 10 bar, 298 K.

Property	Best Pipeline with Parameters
CO_2	XGBRegressor(input_matrix, learning_rate=0.1, max_depth=9, min_child_weight=14, n_estimators=100,n_jobs=1,objective=reg:squarederror,subsample=0.6000000000000001, verbosity=0)
CH_4	ExtraTreesRegressor(input_matrix,bootstrap=False,max_features=0.7000000000000001, min_samples_leaf=1, min_samples_split=5, n_estimators=100)
H_2	RidgeCV(PolynomialFeatures(FastICA(input_matrix,tol=0.8500000000000001),degree=2, include_bias=False, interaction_only=False))
N_2	ExtraTreesRegressor(CombineDFs(input_matrix, input_matrix), bootstrap=False, max_features=0.45, min_samples_leaf=1, min_samples_split=4, n_estimators=100)
O_2	ExtraTreesRegressor(PolynomialFeatures(input_matrix, degree=2, include_bias=False, interaction_only=False), bootstrap=False, max_features=0.45, min_samples_leaf=1, min_samples_split=5, n_estimators=100)

Figures S16-S19 compares the predictions of CoRE+Hypo ML models and simulated gas uptakes for 1,623 COFs. All models achieve R^2 values >0.9 in the test sets and SRCC values close to 1 with very low MAE and RMSE values, showing a very good prediction power. For example, ML-predicted CO_2, CH_4, H_2, N_2 and O_2 uptakes at 1 bar (0.05-12.08, 0.05-7.14, 4×10^{-3}-1.73, 0.02-1.91, 0.03-2.04 mol/kg, respectively) agree very well with simulated values (0.08-14, 0.04-7.61, 5×10^{-3}-1.73, 0.02-1.91, 0.04-1.87 mol/kg, respectively) in **Figure S17** with the accuracy metrics listed in **Table S15**.

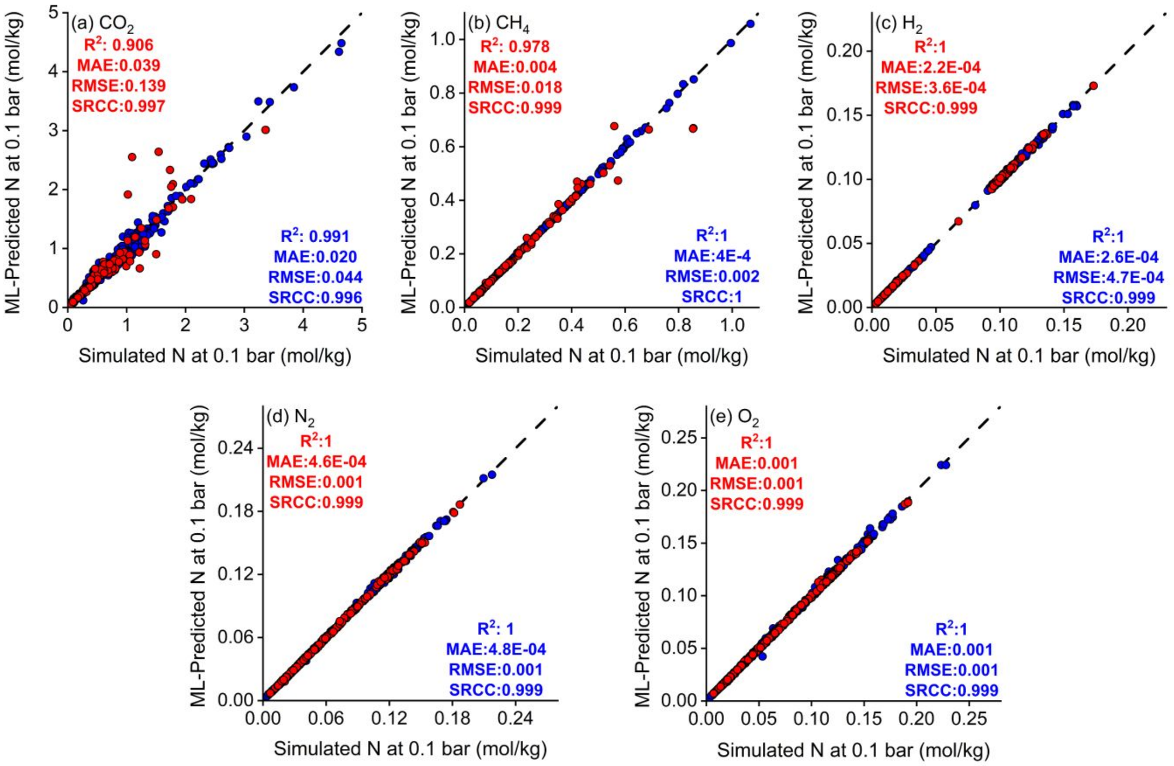

Figure S16. Comparison of ML-predicted and simulated (a) CO_2, (b) CH_4, (c) H_2, (d) N_2, (e) O_2 uptakes of 1,298 COFs in the training set and 325 COFs in the test set at 0.1 bar, 298 K. Blue (red) symbols represent training (test) data.

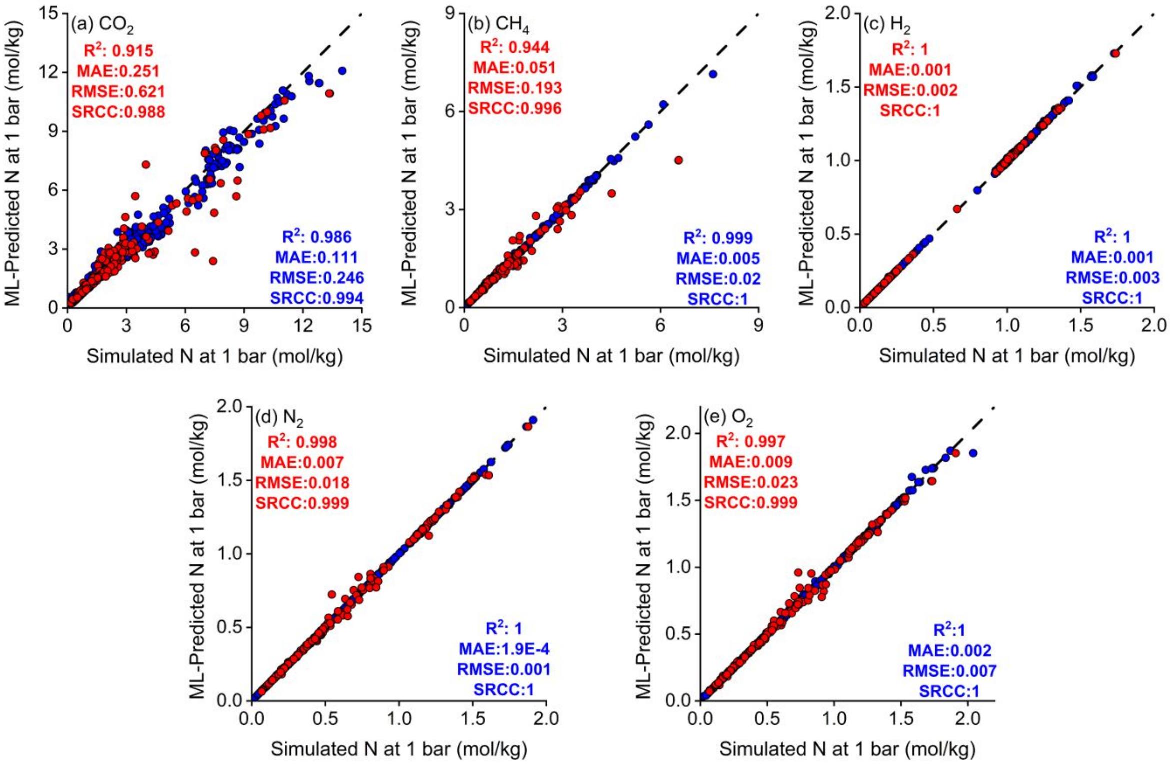

Figure S17. Comparison of ML-predicted and simulated (a) CO_2, (b) CH_4, (c) H_2, (d) N_2, (e) O_2 uptakes of 1,298 COFs in the training set and 325 COFs in the test set at 1 bar, 298 K. Blue (red) symbols represent training (test) data.

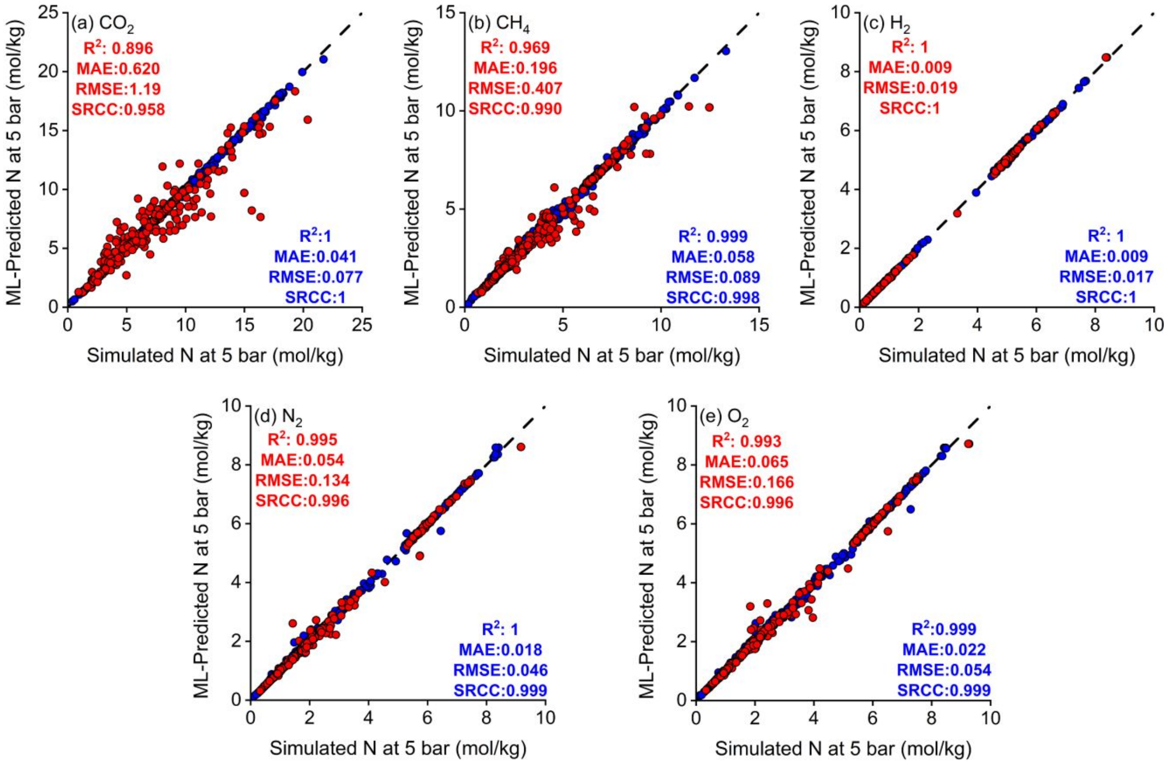

Figure S18. Comparison of ML-predicted and simulated (a) CO_2, (b) CH_4, (c) H_2, (d) N_2, (e) O_2 uptakes of 1,298 COFs in the training set and 325 COFs in the test set at 5 bar, 298 K. Blue (red) symbols represent training (test) data.

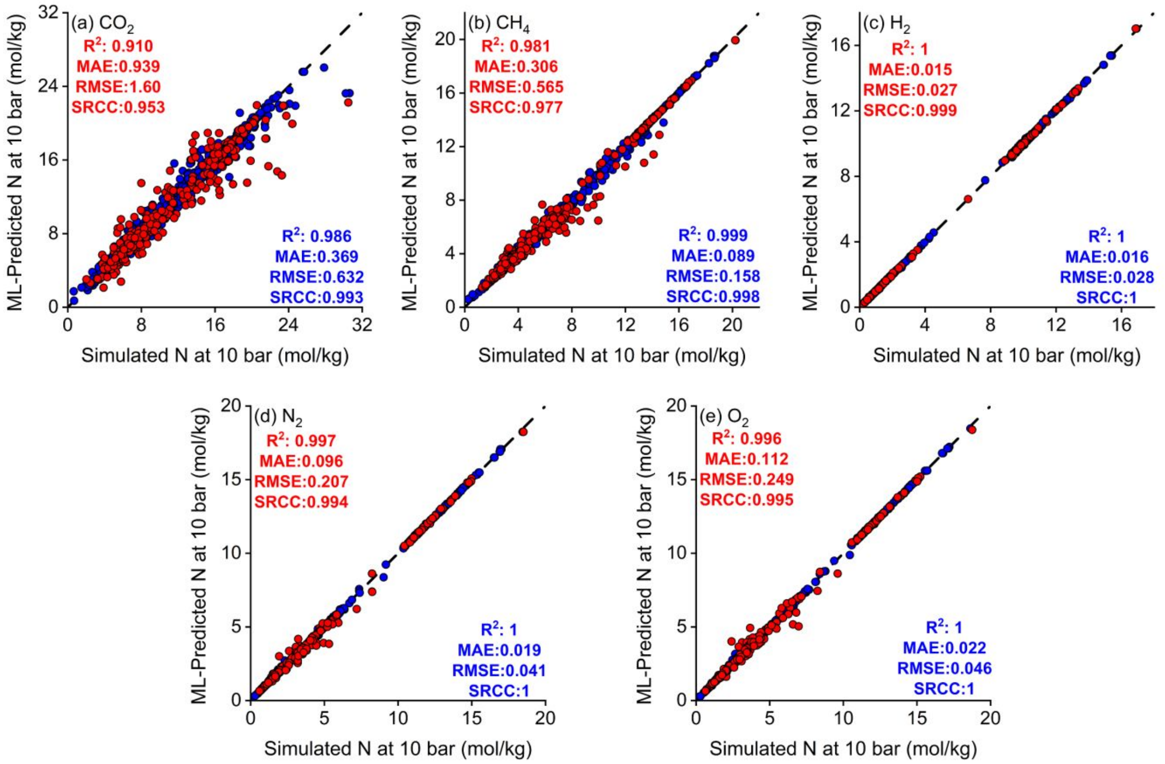

Figure S19. Comparison of ML-predicted and simulated (a) CO_2, (b) CH_4, (c) H_2, (d) N_2, (e) O_2 uptakes of 1,298 COFs in the training set and 325 COFs in the test set at 10 bar, 298 K. Blue (red) symbols represent training (test) data.

Table S15. The statistical accuracy metrics calculated for the CoRE+Hypo ML-predicted gas uptakes for the training and test sets. R^2 and SRCC are dimensionless values ranging from 0 to 1, whereas MAE and RMSE are expressed in units of mol/kg.

Target Data	Training Set	Test Set
CO_2-0.1 bar	R^2:0.991, MAE:0.02, RMSE:0.04, SRCC:0.996	R^2:0.906, MAE:0.039, RMSE:0.139, SRCC:0.997
CO_2-1 bar	R^2:0.986, MAE:0.111, RMSE:0.246, SRCC:0.994	R^2:0.915, MAE:0.251, RMSE:0.621, SRCC:0.988
CO_2-5 bar	R^2:1, MAE:0.041, RMSE:0.077, SRCC:1	R^2:0.896, MAE:0.620, RMSE:1.19, SRCC:0.958
CO_2-10 bar	R^2:0.986, MAE:0.369, RMSE:0.632, SRCC:0.993	R^2:0.910, MAE:0.939, RMSE:1.60, SRCC:0.953
CH_4-0.1 bar	R^2:1, MAE:4E-4, RMSE:0.002, SRCC:1	R^2:0.978, MAE:0.004, RMSE:0.018, SRCC:0.999
CH_4-1 bar	R^2:0.999, MAE:0.005, RMSE:0.02, SRCC:1	R^2:0.944, MAE:0.051, RMSE:0.193, SRCC:0.996
CH_4-5 bar	R^2:0.999, MAE:0.058, RMSE:0.089, SRCC:0.998	R^2:0.969, MAE:0.196, RMSE:0.407, SRCC:0.990
CH_4-10 bar	R^2:0.999, MAE:0.089, RMSE:0.158, SRCC:0.998	R^2:0.981, MAE:0.306, RMSE:0.565, SRCC:0.977
H_2-0.1 bar	R^2:1, MAE:2.6E-4, RMSE:4.7E-4, SRCC:0.999	R^2:1, MAE:2.2E-4, RMSE:3.6E-4, SRCC:0.999
H_2-1 bar	R^2:1, MAE:0.001, RMSE:0.003, SRCC:1	R^2:1, MAE:0.001, RMSE:0.002, SRCC:1
H_2-5 bar	R^2:1, MAE:0.009, RMSE:0.017, SRCC:1	R^2:1, MAE:0.009, RMSE:0.019, SRCC:1
H_2-10 bar	R^2:1, MAE:0.016, RMSE:0.028, SRCC:1	R^2:1, MAE:0.015, RMSE:0.027, SRCC:0.999
N_2-0.1 bar	R^2:1, MAE:4.8E-4, RMSE:0.001, SRCC:0.999	R^2:1, MAE: 4.6E-4, RMSE:0.001, SRCC:0.999
N_2-1 bar	R^2:0.999, MAE:1.9E-4, RMSE:0.001, SRCC:1	R^2:0.998, MAE: 0.007, RMSE:0.018, SRCC:0.999
N_2-5 bar	R^2:1, MAE:0.018, RMSE:0.046, SRCC:0.999	R^2:0.995, MAE: 0.054, RMSE:0.134, SRCC:0.996
N_2-10 bar	R^2:1, MAE:0.019, RMSE:0.041, SRCC:1	R^2:0.997, MAE: 0.096, RMSE:0.207, SRCC:0.994
O_2-0.1 bar	R^2:1, MAE:0.001, RMSE:0.001, SRCC:0.999	R^2:1, MAE:0.001, RMSE:0.001, SRCC:0.999
O_2-1 bar	R^2:1, MAE:0.002, RMSE:0.007, SRCC:1	R^2:0.997, MAE:0.009, RMSE:0.023, SRCC:0.999
O_2-5 bar	R^2:0.999, MAE:0.022, RMSE:0.054, SRCC:0.999	R^2:0.993, MAE:0.065, RMSE:0.166, SRCC:0.996
O_2-10 bar	R^2:1, MAE:0.022, RMSE:0.046, SRCC:1	R^2:0.996, MAE:0.112, RMSE:0.249, SRCC:0.995

Figures S20-S23 show a significant improvement in the agreement between ML-predicted and simulated gas uptakes of 6,309 unseen hypoCOFs. For example, at 1 bar, CoRE+Hypo ML models achieved R^2 of 0.86 for CO_2 and 0.94 for CH_4 as shown in **Figures S21(a-b)**, significantly outperforming the corresponding CoRE models having R^2 values of 0.59 and 0.47, respectively. While CoRE ML models struggled to predict high N_2 and O_2 uptakes at 5 and 10 bar as discussed, CoRE+Hypo ML models consistently achieved R^2 and SRCC values almost 1 for both gases. Thus, we concluded that the CoRE+Hypo ML models are capable of accurately predicting the gas uptakes of both CoRE COFs and unseen hypoCOFs.

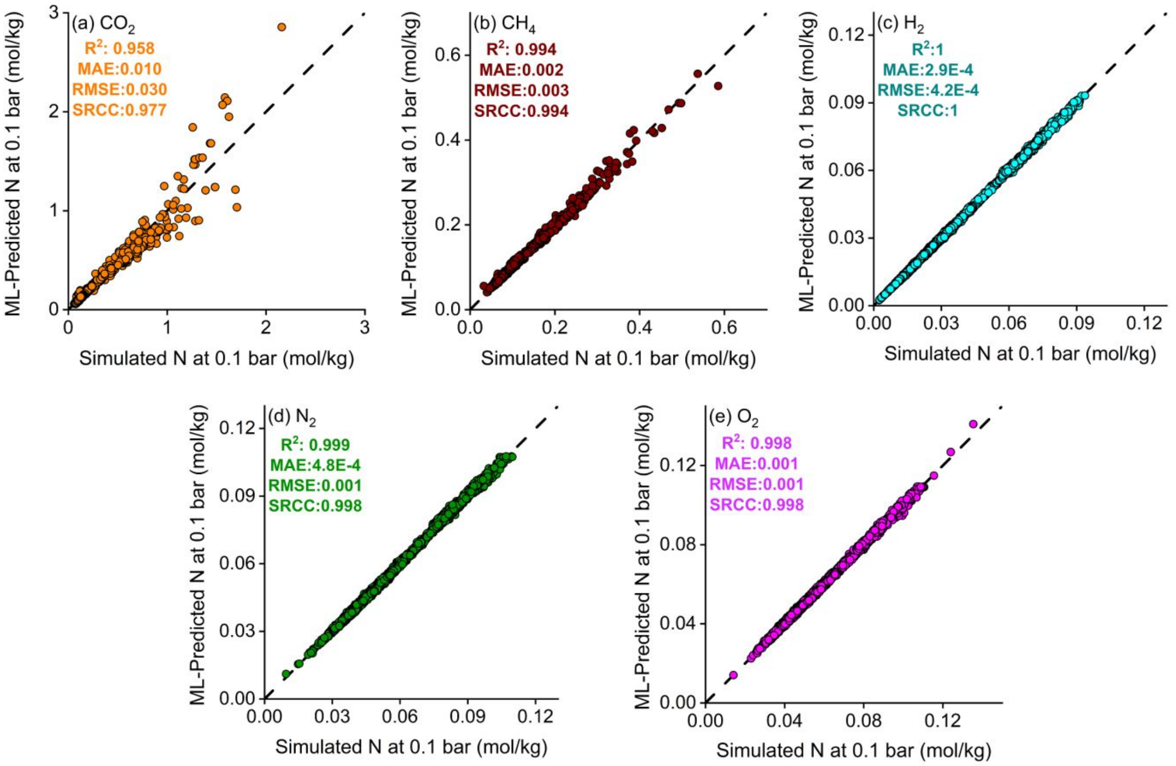

Figure S20. Comparison of ML-predicted and simulated (a) CO_2, (b) CH_4, (c) H_2, (d) N_2, (e) O_2 uptakes of 6,309 unseen hypoCOFs at 0.1 bar, 298 K.

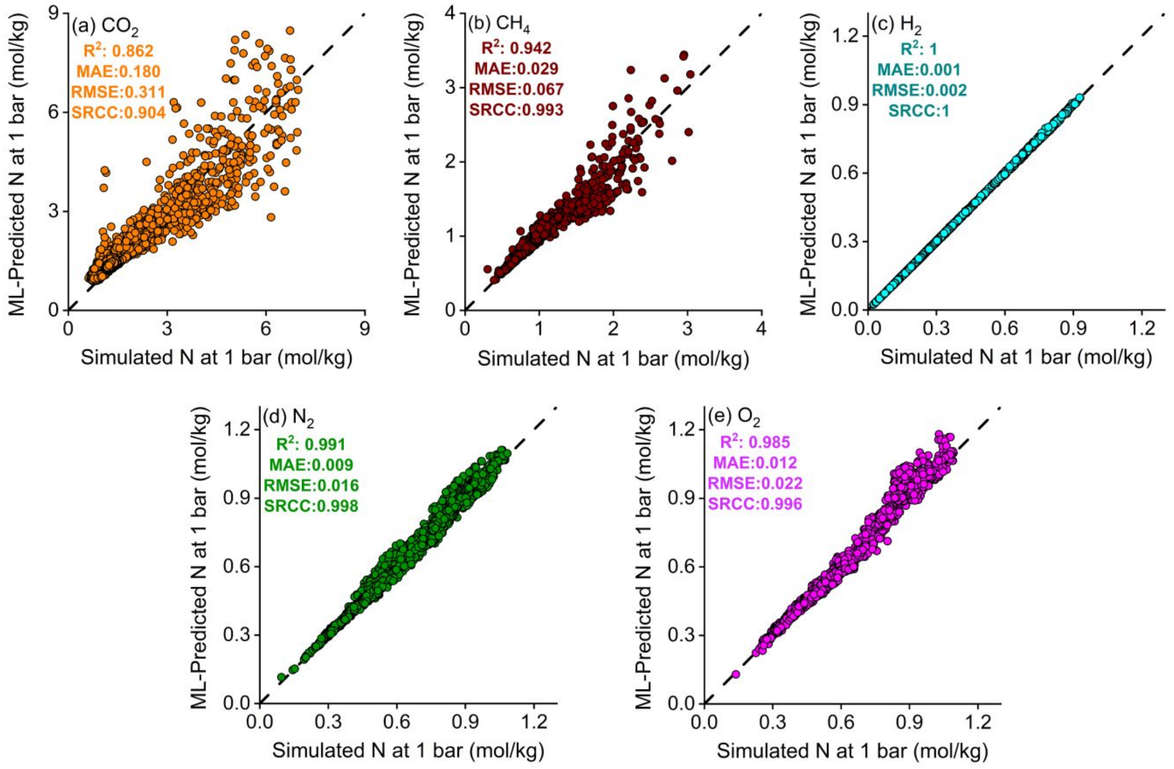

Figure S21. Comparison of ML-predicted and simulated (a) CO_2, (b) CH_4, (c) H_2, (d) N_2, (e) O_2 uptakes of 6,309 unseen hypoCOFs at 1 bar, 298 K.

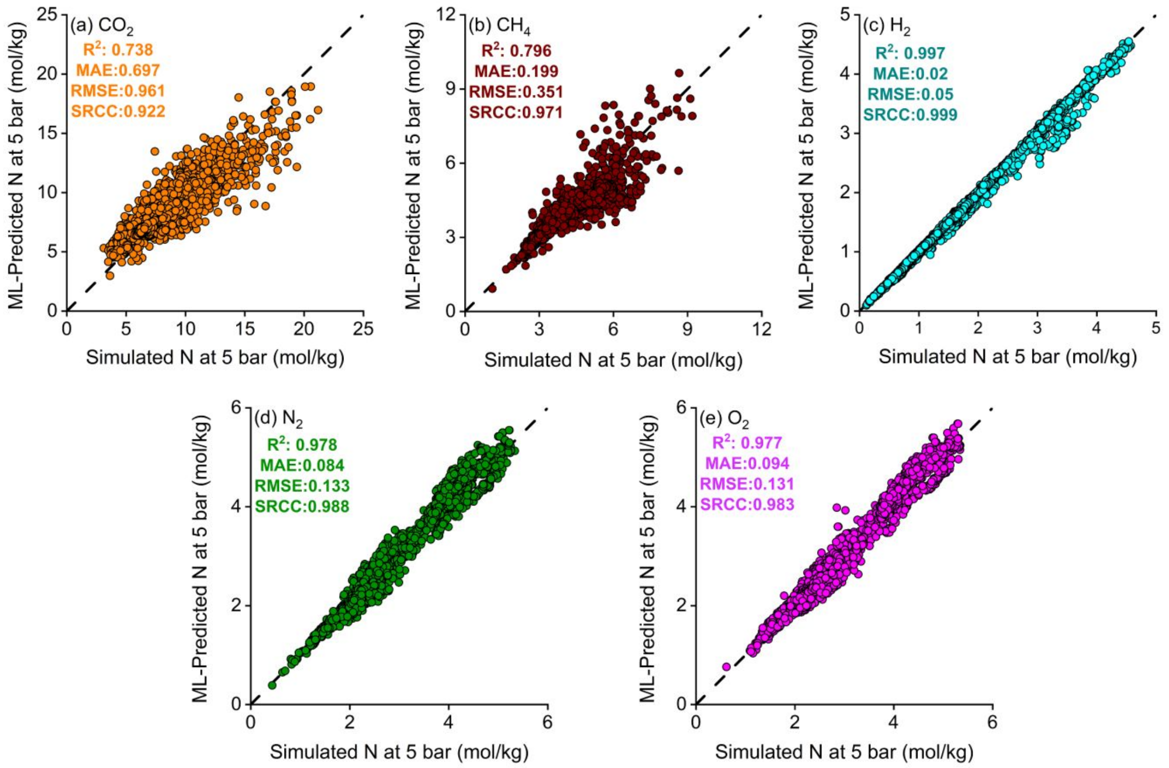

Figure S22. Comparison of ML-predicted and simulated (a) CO_2, (b) CH_4, (c) H_2, (d) N_2, (e) O_2 uptakes of 6,309 unseen hypoCOFs at 5 bar, 298 K.

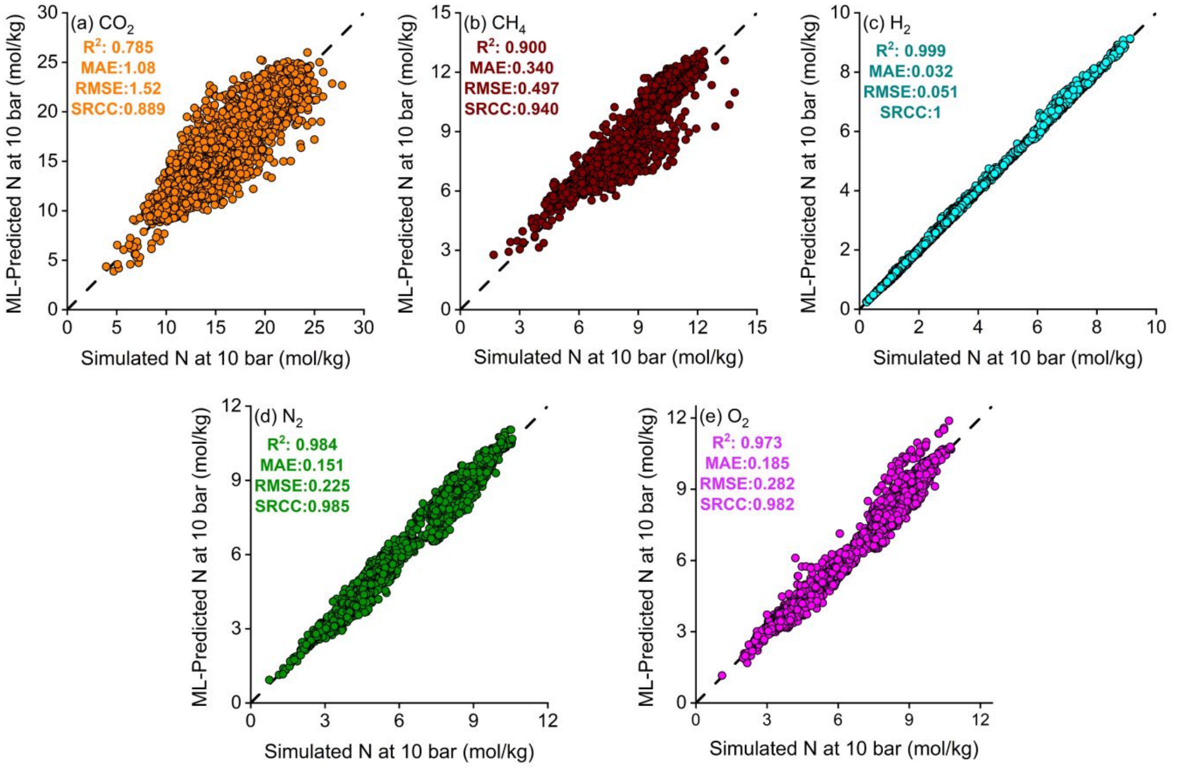

Figure S23. Comparison of ML-predicted and simulated (a) CO_2, (b) CH_4, (c) H_2, (d) N_2, (e) O_2 uptakes of 6,309 unseen hypoCOFs at 10 bar, 298 K.

Table S16. The statistical accuracy metrics calculated for the CoRE+Hypo ML-predicted gas uptakes of 6,309 unseen hypoCOFs. R^2 and SRCC are dimensionless values ranging from 0 to 1, whereas MAE and RMSE are expressed in units of mol/kg.

**Target Data**	**Unseen Set**
CO_2-0.1 bar	R^2:0.958, MAE:0.01, RMSE:0.03, SRCC:0.977
CO_2-1 bar	R^2:0.862, MAE:0.180, RMSE:0.311, SRCC:0.904
CO_2-5 bar	R^2:0.738, MAE:0.697, RMSE:0.961, SRCC:0.922
CO_2-10 bar	R^2:0.785, MAE:1.081, RMSE:1.515, SRCC:0.889
CH_4-0.1 bar	R^2:0.994, MAE:0.002, RMSE:0.003, SRCC:0.994
CH_4-1 bar	R^2:0.942, MAE:0.029, RMSE:0.067, SRCC:0.993
CH_4-5 bar	R^2:0.796, MAE:0.199, RMSE:0.351, SRCC:0.971
CH_4-10 bar	R^2:0.900, MAE:0.340, RMSE:0.497, SRCC:0.940
H_2-0.1 bar	R^2:1, MAE:2.9E-04, RMSE:4.2E-4, SRCC:1
H_2-1 bar	R^2:1, MAE:0.001, RMSE:0.002, SRCC:1
H_2-5 bar	R^2:0.997, MAE:0.02, RMSE:0.05, SRCC:0.999
H_2-10 bar	R^2:0.999, MAE:0.03, RMSE:0.05, SRCC:1
N_2-0.1 bar	R^2:0.999, MAE:4.8E-4, RMSE:0.001, SRCC:0.999

N_2-1 bar	R^2:0.991, MAE:0.01, RMSE:0.02, SRCC:0.998
N_2-5 bar	R^2:0.978, MAE:0.08, RMSE:0.133, SRCC:0.988
N_2-10 bar	R^2:0.984, MAE:0.151, RMSE:0.225, SRCC:0.985
O_2-0.1 bar	R^2:0.998, MAE:0.001, RMSE:0.001, SRCC:0.998
O_2-1 bar	R^2:0.985, MAE:0.012, RMSE:0.022, SRCC:0.996
O_2-5 bar	R^2:0.977, MAE:0.094, RMSE:0.131, SRCC:0.982
O_2-10 bar	R^2:0.973, MAE:0.185, RMSE:0.282, SRCC:0.982

5. Comparison of the COF Space with Other Porous Materials:

For gas storage applications, a recent review compiled experimentally measured CO_2 uptakes of various COFs, ranging from 0.4 to 74.3 cm^3/g at 1 bar and 298 K.[28] We identified 71 CoRE COFs and 1,361 hypoCOFs surpassing this range under the same conditions. In another review, experimentally measured CH_4 uptakes of COFs were reported in between 8-32 cm^3/g at 1 bar, 298 K.[29] In COF Space, we discovered 661 hypoCOFs and 30 CoRE COFs achieving higher CH_4 uptakes than these values. To the best of our knowledge, there is no existing data on H_2, N_2 and O_2 adsorption data of COFs under the conditions we investigated in this work, which represents the contribution of our models into the field. We also compared COFs with MOFs which exhibit CO_2 uptakes ranging from 22.9 to 136.9 cm^3/g at 1 bar, 298 K.[30] Within COF Space, 203 hypoCOFs and 12 CoRE COFs have CO_2 uptakes exceeding 136.9 cm^3/g. Several prominent MOFs, HKUST-1, Ni-MOF-74, PCN-14, UTSA-20, NU-125 and NU-111 were reported to achieve CH_4 uptakes in the range of ~11.5-26 mg/g and ~80.7-110 mg/g at 1 and 10 bar, respectively.[31] COFs demonstrated CH_4 uptakes up to 114.6 and 320 mg/g at 1 and 10 bar, respectively.

For gas separations, many COFs achieve higher CO_2/H_2 selectivities (1.2-171) compared to experimentally studied COF NUS-2 (117),[32] zeolites FAU (17),[33] and CHA (117)[33] at 1 bar. For CH_4/H_2 separation, COFs' selectivities (1.2-91.9 at 1 bar, and 1.2-16.2 at 10 bar) are comparable to simulated selectivities of MOFs (1-100 at 1 bar, and 1-20 at 10 bar).[34] CO_2/CH_4 selectivity range of COFs (1-17.5) is similar to experimentally reported selectivities of CTF-DI series (5-12),[35] AzoCOF series (5.2-37),[36] and ND-COF series(~2),[37] and higher than those of MOR (10 at 1 bar)[38] and NaX (40).[33] For CH_4/N_2 and O_2/N_2 separation, COFs are not promising adsorbents due to their low selectivities.

References

(1) Willems, T. F.; Rycroft, C. H.; Kazi, M.; Meza, J. C.; Haranczyk, M. Algorithms and Tools for High-Throughput Geometry-Based Analysis of Crystalline Porous Materials. *Microporous Mesoporous Mater.* **2012**, *149*, 134-141.
(2) Wilmer, C. E.; Snurr, R. Q. Towards Rapid Computational Screening of Metal-Organic Frameworks for Carbon Dioxide Capture: Calculation of Framework Charges via Charge Equilibration. *Chem. Eng. J.* **2011**, *171*, 775-781.
(3) Dubbeldam, D.; Calero, S.; Ellis, D. E.; Snurr, R. Q. RASPA: Molecular Simulation Software for Adsorption and Diffusion in Flexible Nanoporous Materials. *Mol. Simul.* **2016**, *42*, 81-101.
(4) Mayo, S. L.; Olafson, B. D.; Goddard, W. A. DREIDING: A Generic Force Field for Molecular Simulations. *J. Phys. Chem.* **1990**, *94*, 8897-8909.
(5) Potoff, J. J.; Siepmann, J. I. Vapor–Liquid Equilibria of Mixtures Containing Alkanes, Carbon Dioxide, and Nitrogen. *AIChE J.* **2001**, *47*, 1676-1682.
(6) Martin, M. G.; Siepmann, J. I. Transferable Potentials for Phase Equilibria. 1. United-Atom Description of n-alkanes. *J. Phys. Chem. B* **1998**, *102*, 2569-2577.
(7) Buch, V. Path Integral Simulations of Mixed Para-D2 and Ortho-D2 Clusters: The Orientational Effects. *J. Chem. Phys.* **1994**, *100*, 7610-7629.
(8) Makrodimitris, K.; Papadopoulos, G. K.; Theodorou, D. N. Prediction of Permeation Properties of CO_2 and N_2 through Silicalite via Molecular Simulations. *J. Phys. Chem. B* **2001**, *105*, 777-788.
(9) Mellot, C.; Lignieres, J. Monte Carlo Simulations of N_2 and O_2 Adsorption in Silicalite and CaLSX Zeolites. *Mol. Simul.* **1997**, *18*, 349-365.
(10) Aksu, G. O.; Daglar, H.; Altintas, C.; Keskin, S. Computational Selection of High-Performing Covalent Organic Frameworks for Adsorption and Membrane-Based CO_2/H_2 Separation. *J. Phys. Chem. C* **2020**, *124*, 22577-22590.
(11) Altundal, O. F.; Altintas, C.; Keskin, S. Can COFs Replace MOFs in Flue Gas Separation? High-Throughput Computational Screening of COFs for CO_2/N_2 Separation. *J. Mater. Chem. A* **2020**, *8*, 14609-14623.
(12) Altundal, O. F.; Haslak, Z. P.; Keskin, S. Combined GCMC, MD, and DFT Approach for Unlocking the Performances of COFs for Methane Purification. *Ind. Eng. Chem. Res.* **2021**, *60*, 12999-13012.
(13) Yan, T.; Lan, Y.; Tong, M.; Zhong, C. Screening and Design of Covalent Organic Framework Membranes for CO_2/CH_4 Separation. *ACS Sustain. Chem. Eng.* **2018**, *7*, 1220-1227.
(14) Ongari, D.; Yakutovich, A. V.; Talirz, L.; Smit, B. Building a Consistent and Reproducible Database for Adsorption Evaluation in Covalent–Organic Frameworks. *ACS Cent. Sci.* **2019**, *5*, 1663-1675.
(15) Ewald, P. P. Evaluation of Optical and Electrostatic Lattice Potentials. *Ann. Phys.* **1921**, *369*, 253-287.
(16) Frenkel, D.; Smit, B. *Understanding Molecular Simulation: From Algorithms to Applications*; Elsevier, 2023.
(17) Tong, M.; Lan, Y.; Yang, Q.; Zhong, C. Exploring the Structure-Property Relationships of Covalent Organic Frameworks for Noble Gas Separations. *Chem. Eng. Sci.* **2017**, *168*, 456-464.
(18) Mercado, R.; Fu, R.-S.; Yakutovich, A. V.; Talirz, L.; Haranczyk, M.; Smit, B. In Silico Design of 2D and 3D Covalent Organic Frameworks for Methane Storage Applications. *Chem Mater* **2018**, *30*, 5069-5086.
(19) Le, T. T.; Fu, W.; Moore, J. H. Scaling Tree-Based Automated Machine Learning to Biomedical Big Data with a Feature Set Selector. *Bioinformatics* **2020**, *36*, 250-256.
(20) Martinsson, P.-G.; Rokhlin, V.; Tygert, M. A Randomized Algorithm for the Decomposition of Matrices. *Appl. Comput. Harmon. Anal.* **2011**, *30*, 47-68.
(21) Meduri, S.; Nandanavanam, J. Prediction of Hydrogen Uptake of Metal Organic Frameworks Using Explainable Machine Learning. *Energy and AI* **2023**, *12*, 100230.
(22) Dureckova, H.; Krykunov, M.; Aghaji, M. Z.; Woo, T. K. Robust Machine Learning Models for Predicting High CO_2 Working Capacity and CO_2/H_2 Selectivity of Gas Adsorption in Metal Organic Frameworks for Precombustion Carbon Capture. *J. Phys. Chem. C* **2019**, *123*, 4133-4139.
(23) Liang, H.; Jiang, K.; Yan, T.-A.; Chen, G.-H. XGBoost: An Optimal Machine Learning Model with Just Structural Features to Discover MOF Adsorbents of Xe/Kr. *ACS Omega* **2021**, *6*, 9066-9076.

(24) Ogutu, J. O.; Schulz-Streeck, T.; Piepho, H.-P. Genomic Selection Using Regularized Linear Regression Models: Ridge Regression, Lasso, Elastic Net and Their Extensions. In *BMC Proc.*, 2012; Springer: Vol. 6, pp 1-6.
(25) Calvetti, D.; Morigi, S.; Reichel, L.; Sgallari, F. Tikhonov Regularization and the L-Curve for Large Discrete Ill-Posed Problems. *J. Comput. Appl. Math.* **2000**, *123*, 423-446.
(26) Fanourgakis, G. S.; Gkagkas, K.; Froudakis, G. Introducing Artificial MOFs for Improved Machine Learning Predictions: Identification of Top-Performing Materials for Methane Storage. *J. Chem. Phys.* **2022**, *156*.
(27) Aksu, G. O.; Keskin, S. Rapid and Accurate Screening of the COF Space for Natural Gas Purification: COFInformatics. *ACS Appl. Mater. Interfaces* **2024**, *16*, 19806-19818.
(28) Li, H.; Dilipkumar, A.; Abubakar, S.; Zhao, D. Covalent Organic Frameworks for CO_2 Capture: From Laboratory Curiosity to Industry Implementation. *Chem. Soc. Rev.* **2023**, *52*, 6294-6329.
(29) Xue, S.; Ma, X.; Wang, Y.; Duan, G.; Zhang, C.; Liu, K.; Jiang, S. Advanced Development of Three-Dimensional Covalent Organic Frameworks: Valency Design, Functionalization, and Applications. *Coord. Chem. Rev.* **2024**, *504*, 215659.
(30) Fan, W.; Zhang, X.; Kang, Z.; Liu, X.; Sun, D. Isoreticular Chemistry within Metal–Organic Frameworks for Gas Storage and Separation. *Coord. Chem. Rev.* **2021**, *443*, 213968.
(31) Peng, Y.; Krungleviciute, V.; Eryazici, I.; Hupp, J. T.; Farha, O. K.; Yildirim, T. Methane Storage in Metal–Organic Frameworks: Current Records, Surprise Findings, and Challenges. *J. Am. Chem. Soc.* **2013**, *135*, 11887-11894.
(32) Kang, Z.; Peng, Y.; Qian, Y.; Yuan, D.; Addicoat, M. A.; Heine, T.; Hu, Z.; Tee, L.; Guo, Z.; Zhao, D. Mixed Matrix Membranes (MMMs) Comprising Exfoliated 2D Covalent Organic Frameworks (COFs) for Efficient CO_2 Separation. *Chem. Mat.* **2016**, *28*, 1277-1285.
(33) Krishna, R.; van Baten, J. M. In Silico Screening of Metal–Organic Frameworks in Separation Applications. *Phys. Chem. Chem. Phys.* **2011**, *13*, 10593-10616.
(34) Altintas, C.; Erucar, I.; Keskin, S. High-Throughput Computational Screening of the Metal Organic Framework Database for CH_4/H_2 Separations. *ACS Appl. Mater. Interfaces* **2018**, *10*, 3668-3679.
(35) Du, J.; Cui, Y.; Liu, Y.; Krishna, R.; Yu, Y.; Wang, S.; Zhang, C.; Song, X.; Liang, Z. Preparation of Benzodiimidazole-Containing Covalent Triazine Frameworks for Enhanced Selective CO_2 Capture and Separation. *Microporous Mesoporous Mater.* **2019**, *276*, 213-222.
(36) Huang, S.; Hu, Y.; Tan, L.-L.; Wan, S.; Yazdi, S.; Jin, Y.; Zhang, W. Highly C_2/C_1-Selective Covalent Organic Frameworks Substituted with Azo Groups. *ACS Appl. Mater. Interfaces* **2020**, *12*, 51517-51522.
(37) Kumar, S.; Abdulhamid, M. A.; Wonanke, A. D.; Addicoat, M. A.; Szekely, G. Norbornane-Based Covalent Organic Frameworks for Gas Separation. *Nanoscale* **2022**, *14*, 2475-2481.
(38) García-Pérez, E.; Parra, J.; Ania, C.; García-Sánchez, A.; Van Baten, J.; Krishna, R.; Dubbeldam, D.; Calero, S. A Computational Study of CO_2, N_2, and CH_4 Adsorption in Zeolites. *Adsorption* **2007**, *13*, 469-476.
